# Transcription factor ZNF22 regulates blood-tumor barrier permeability by interacting with HDAC3 protein

**DOI:** 10.3389/fnmol.2022.1027942

**Published:** 2022-11-28

**Authors:** Baicheng Zhu, Lu Zhang, Xinxin Zhou, Hao Ning, Teng Ma

**Affiliations:** ^1^Department of Neurobiology, School of Life Sciences, China Medical University, Shenyang, China; ^2^Liaoning TCM Academy, Liaoning University of Traditional Chinese Medicine, Shenyang, China

**Keywords:** ZNF22, HDAC3, BTB, glioma, protein interactions

## Abstract

**Objective:**

The primary goals of this study were to investigate the potential roles of ZNF22 and HDAC3 as a histone deacetylase in regulating an increases in blood-tumor barrier (BTB) permeability and some of the possible molecular mechanisms associated with this effect.

**Methods:**

The expression of ZNF22 and HDAC3 in glioma-exposed endothelial cells (GECs) of BTB were detected transcription real-time PCR or western blot. The interaction of ZNF22 and HDAC3 in GECs associated with transcript effect was analyzed by means of Co-Immunoprecipitation and luciferase reporter assay.

**Results:**

In the present investigation, GECs expressed higher levels of ZNF22 as a zinc finger transcription factor and HDAC3 than endothelial cells. We then affirmed that silencing HDAC3 or ZNF22 led to a reduction in BTB permeability. By bioinformatics analysis, chromatin immunoprecipitation (ChIP) assays and luciferase assay, we found that ZNF22 had a target binding relationship with the promoter regions of ZO-1, Occludin, and Claudin-5 and negatively regulated the expression of ZO-1, Occludin, and Claudin-5. Furthermore, we revealed that HDAC3, as a co-transcript repressor with histone deacetylase activity, could interact with ZNF22 to hinder the expression of TJ-associated proteins, thereby further facilitating the permeability of BTB.

**Conclusion:**

ZNF22 acted as a transcription factor in conjunction with HDAC3 to modulate the expression of TJ-associated proteins, which was correlated with an increase in BTB permeability. These results may provide new strategies and targets for the chemotherapy of gliomas as well as intracranial infections.

## Introduction

Primary malignant gliomas are the most common type of brain tumor found in adults (McNeill, 2016; Lapointe et al., 2018). 25.1% of all primary brain tumors and other central nervous system (CNS) cancers are gliomas, while 80.8% of all malignant tumors are gliomas (Ostrom et al., 2020). Due to its biological characteristics, the treatment of glioma is complicated. First, due to the particularity of its location and highly invasive, the difficulty of surgery is considerably increased (Aldape et al., 2019). Second, the presence of the blood-brain barrier (BBB) poses a huge challenge in developing drugs to treat brain tumors (Zhao et al., 2020).

Surgery is still the main therapy option for early-stage gliomas, and the principle is to remove as much of the tumor as possible, while preserving the maximum amount of normal tissue (Woernle et al., 2015). Nevertheless, most gliomas are infiltrative in nature and do not have a clear histological boundary with normal brain tissue, making it difficult to gain a true biological total resection (Zhang et al., 2021).

Infections of the CNS provide difficulties in treatment that are similar to those presented by CNS tumors. Due to the existence of the BBB, the concentration of medications within the central nervous system (CNS) is restricted during therapy for CNS infections; thus, the dosage of systemic treatments needs to be raised in order to achieve successful treatment (Nau et al., 2010). For example, increasing the dose of cefotaxime from 6 g/day to 24 g/day substantially improved efficacy when the meningitis was caused by L. pneumoniae with reduced susceptibility to broad-spectrum cephalosporins (Viladrich et al., 1996). Therefore, increasing the permeability of the BBB improved the entry of medications into brain tissue and cerebrospinal fluid, enabling them to reach effective concentrations more quickly. This was made possible by the fact that the BBB was made more permeable.

The BBB is a combination of physiological properties of endothelial cells (ECs) that restricts vascular permeability (Profaci et al., 2020). BBB is mostly made up of cerebral microvascular endothelial cells, pericytes, extracellular matrix, and perivascular astrocytes’ foot processes (Wood, 2010; Daneman and Prat, 2015). The tight junction (TJ) between cerebral endothelial cells, choroid plexus epithelial cells and arachnoid epithelial cells is an important characteristic structure of BBB (Abbott et al., 2010; Yang et al., 2019). The BBB plays several critical functions in the CNS (Deligne et al., 2020). The BBB is disrupted during tumor development and is called the blood tumor barrier (BTB). At the ultrastructural level, changes in the BTB include a rise in the number of endothelial vesicles, an increase in endothelial growth, and a widening of the TJ, all of which point to more *cis*-cell and cross-cell transport. Despite the fact that the BTB is more permeable than the BBB, its variable permeability to small and big molecules and heterogeneous perfusion result in inefficient medication concentration in gliomas (Arvanitis et al., 2020; Hempel et al., 2020).

ZNF22, as a zinc finger protein located in the 10q11.2, is a classical transcription factor. ZNF22 was reported to be associated with the prognosis of glioma (Cheng et al., 2019). In our earlier KEGG analysis, we found that ZNF22 was negatively correlated with the expression of gap junctions, and it was indicated that gap junctions could also be involved in the barrier function of the BBB (Deli, 2009).

Histone deacetylase (HDAC) is an enzyme that removes the acetyl group from the NH2-terminal lysine residue of core histones, resulting in a more closed chromatin structure and thus inhibiting gene expression (Lai et al., 2012). It was determined that activated HDAC decreased TJ nsprotein expression, but HDAC inhibitor (HDACi) treatment increased the production of TJ-associated proteins (Bordin et al., 2004). It was shown that HDAC3 was an epigenetic drug target that was currently being labeled as a potential therapeutic strategy against multiple cancers (Sarkar et al., 2020). In addition, its homologue HDAC1 promoted the permeability of the BBB by inhibiting the expression of CLDN5 (Dudek et al., 2020).

Through the JASPER,^1^ we identified potential sites for binding to ZNF22 in the promoter regions of ZO-1, Occludin, and Claudin-5, suggesting that ZNF22 might be a potential candidate for the transcription factors for TJ-related proteins. Furthermore, bioinformatic tools GeneMAINA^2^ and String^3^ predicted binding relationship between ZNF22 and HDAC3, speculating that the interaction between ZNF22 and HDAC3 might regulate BTB permeability. Predicted results in pictures were shown in Supplementary Figure 1A.

We would like to offer a unique strategy for CNS infection and glioma therapy based on our study of the association between ZNF22, HDAC3, TJ-associated proteins, and BBB/BTB permeability. Initially, the expression and function of these factors in glioma-exposed BBB endothelial cells (GECs) will be comprehensively described and investigated. Then, the regulatory mechanisms of TJ-associated protein expression and BBB/BTB permeability governed by the relationship between ZNF22 and HDAC3 will be investigated. We want to elucidate a novel signaling mechanism for the treatment of CNS infections and gliomas.

## Materials and methods

### Bioinformatics data download and processing

The datasets of ZNF22 and HDAC3 expression in glioma and paired normal tissue were downloaded from TCGA database of GDC.^4^ Visualization of the data was analysed by means of the R software package “limma” for variance analysis and the R software package “beeswarm” and “survival”. DAVID Functional Annotation Bioinformatics Microarray Analysis (ncifcrf.gov) was used to perform GO analysis of ZNF22 and HDAC family in glioma, the visualized the data was analysed by the use of R’s ggplot2 package.

### Cell lines and cell culture

This experiment utilized the human microvascular endothelial cell line hCMEC/D3, human U251 glioma cells, human normal astrocytes (NHA). The origin of all cell lines has been described in our previous studies (He et al., 2020; Liu et al., 2020). Furthermore, all cells were cultured in the same manner as in previous studies (He et al., 2020; Liu et al., 2020).

### Construction of blood-brain barrier and blood tumor barrier models *in vitro*

To generate *in vitro* BBB and BTB models, HCMEC/D3 cells were co-cultured with human astrocytes cells and human glioma U251 cells. These cells are termed as ECs (endothelial cells co-cultured with astrocytes) and GECs (glioma-exposed endothelial cells). Details can be found in prior studies (He et al., 2020).

### Quantitative reverse real-time PCR (qRT-PCR)

Details can be found in prior studies (He et al., 2020). The primers that were utilized in this study are detailed in Supplementary Table 1A.

### Cell transfection

Single cell suspensions were prepared by collecting cells in logarithmic growth phase, inoculating the cells in 24-well plates, and transfecting them when they reached 50% to 60% fusion. On pGPU6/GFP/Neo vector (GenePharma, Shanghai, China), ZNF22(–) and HDAC3(–) knockdown plasmids were constructed and designated accordingly. The non-targeting sequences were utilized as NC groups, respectively. Stably transfecting the aforementioned plasmids into GECs required the use of LTX and Plus (Life Technologies, Carlsbad, CA, USA). Using G418 to screen for consistently transfected cells. Supplementary Table 1B lists the primers that were employed in this study.

### Transendothelial electric resistance assay

Following the development of the *in vitro* BBB/BTB model, TEER values were determined using a Millicell-ERS (Millipore, Billerica, MA, USA) instrument in accordance with previous literature (Guo et al., 2019). The Millicell-ERS instrument was used to measure the resistance of ECs/GECs monolayers cultured on transwell filters. The surface area of the transwell insert was used to calculate electrical resistance in units of Ω⋅cm^2^.

### Flux assay for horseradism peroxidase

Details can be found in prior studies (He et al., 2020).

### Western blot assay

Based on our previous research, we used protein extraction and quantification (Guo et al., 2019). Protein concentrations were calculated using the bicinchoninic acid technique (Beyotime Institute of Biotechnology, China). SDS-PAGE electrophoresis (Beyotime Institute of Biotechnology, China) was used to separate protein samples. The films were transferred at 100 V, 120 mA current, and 90–120 min. It was then sealed for 2 h in a sealant (5 percent skimmed milk). The sealant was rinsed with TTBS, and the primary antibody diluent was used to dilute the primary antibody: ZNF22 1:1000 and HDAC3 1:1000 (proteintech, Beijing, China), ZO-1 1:300 and Occludin 1:200 and Claudin-5 1:500 (Thermo Scientific, Beijing, China) in certain proportions, sealed in film and left overnight at 4°C. Following three washes with TTBS, the appropriate secondary antibody was added, and the mixture was then incubated for 2 h at room temperature. After three washes with TTBS, luminescence with ECL (Beyotime Institute of Biotechnology, China) was conducted, photographed, and quantified using ImageJ software.

### Immunofluorescence assay

Endothelial cell proteins were stained with the relevant antibodies, followed by incubation with cy3-labeled goat anti-rabbit fluorescent secondary antibody, staining with DAPI, and images were obtained in a Nikon C2 Plus microscope (Nikon, Tokyo, Japan) using C2 plus software, with the same conditions of exposure and excitation for all images (DAPI: Emission wavelength 447.0, Excitation wavelength 408.0, Pinhole radius 60, Alx546: Emission wavelength 585.0, Excitation wavelength 543.5, Pinhole radius 60), and randomly selected images in each group were compared.

Antibody dilution: ZO-1 (1:50; Thermo Scientific, Beijing, China), Occludin (1:50; Thermo Scientific, Beijing, China), Claudin-5 (1:20; Thermo Scientific, Beijing, China). Details are provided in our prior studies (He et al., 2020; Ning et al., 2022). ZO-1, Occludin, and Claudin-5 are red. Nuclei are blue. Scale bar = 30 μm.

### Chromatin immunoprecipitation and ChIP-qPCR analysis

Details are provided in our prior studies (Ning et al., 2022). For the ChIP-qPCR assay, qRT-PCR was used to quantify the ChIP-enriched DNA as previously described in 2.4. The data were normalized to the input. Supplementary Table 1C lists the primers used in this work.

### Reporter vectors construction and dual-luciferase reporter assay

The putative ZNF22-binding promoter region in ZO-1, Occludin, and claudin-5 was detected and validated by a dual luciferase reporter system in GECs, as described in our previous article (Liu et al., 2020). The related sequences are shown in Supplementary Table 1D.

### Co-Immunoprecipitation

The ECs were lysed according to the Co-Immunoprecipitation Kit instructions (Co-IP Kit, BersinBio, Guangzhou, China). To obtain precipitated ZNF22 and HDAC3 immunocomplexes, protein A/G-MagBeads were coupled with anti-ZNF22 and anti-HDAC3 antibodies overnight at 4°C. A non-specific IgG antibody precipitated the complexes as a control. We used Western blot to examine the protein expression obtained by immunoprecipitation with anti-ZNF22 or anti-HDAC3 antibodies. The Input and IgG groups were used as positive and negative controls in the experiment.

### Statistical analysis

It was utilized either GraphPad Prism v8.0 or SPSS 20 to examine all the data. The following statistical analysis was carried out: All data were presented as the mean standard deviation of at least three separate studies; *t*-tests were performed to compare groups, multiple groups were evaluated with one-way ANOVAs, and Shapiro–Wilk tests were used to determine whether the data were normal or lognormal (*P* > 0.05). The data for all bar and scatter plots of the images in the article were verified to be normally distributed (*P* > 0.05). Statistics were deemed significant at *P* < 0.05.

## Results

### ZNF22 was up-regulated in glioma-exposed endothelial cells and increased blood tumor barrier permeability

**FIGURE 1:**
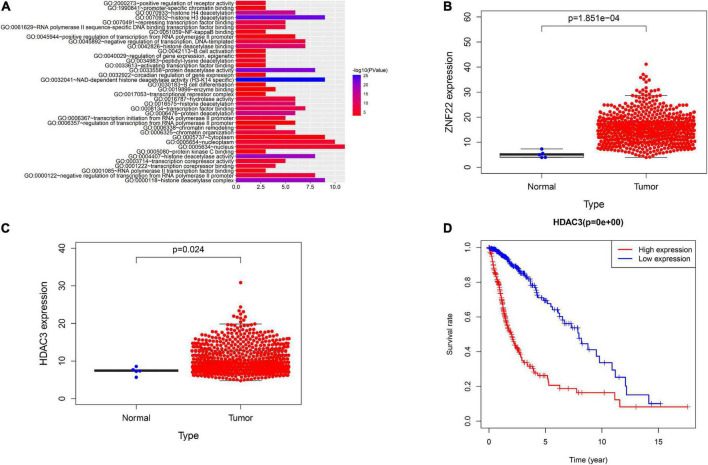
Analysis using bioinformatics. **(A)** GO analysis of HDAC family and ZNF22. **(B)** GDC database-based study of the relative expression of ZNF22 in gliomas. **(C)** GDC database-based study of the relative expression of HDAC3 in gliomas. **(D)** Survival curves of glioma patients with high HDAC3 expression based on the GDC database.

**FIGURE 2:**
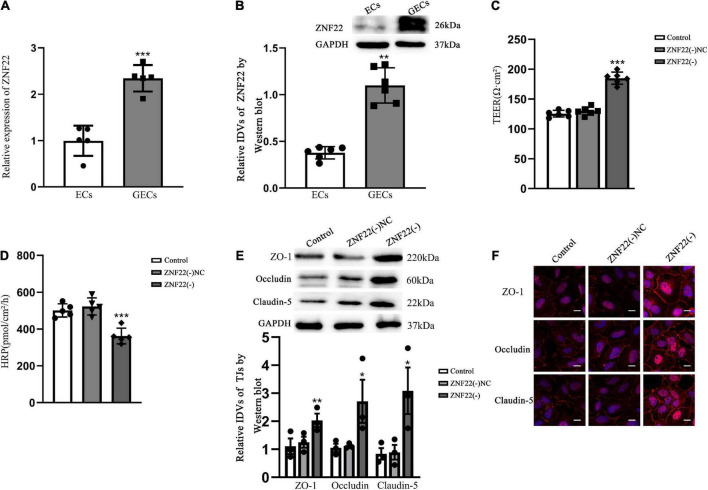
Endogenous expression of ZNF22 was detected in GECs of BTB and was involved in the regulation of BTB permeability *in vitro*. **(A)** qRT-PCR was used to identify the relative expression of ZNF22 in ECs and GECs. As an endogenous control, GAPDH protein levels were measured. Data represent mean ± SD (*n* = 3, each). ***p < 0.001 vs. ECs group. **(B)** Test for the expression of ZNF22 protein using immunoblotting. As an endogenous control, GAPDH protein levels were measured. Data represent mean ± SD (*n* = 3, each). **p < 0.01 vs. ECs group. **(C)** TEER values of GECs were expressed as Ωcm^2^. Data were expressed as mean ± SD (*n* = 3, each). ***p < 0.001 vs. ZNF22(–) NC group. **(D)** pmol/cm^2^/h was used to compute the HRP flow. Data were expressed as mean ± SD (*n* = 3, each). ***p < 0.001 vs. ZNF22(–) NC group. **(E)** TJ-associated proteins ZO-1, Occludin, and Claudin-5 in GECs were examined by protein blotting. As an endogenous control, GAPDH protein levels were measured. Data were expressed as. *p < 0.05 and **p < 0.01 vs. ZNF22(–) NC group. **(F)** ZO-1, Occludin, and Claudin-5 immunofluorescent localization in GECs. ZO-1, Occludin, and Claudin-5 all were colored red using fluorescent secondary antibodies, and DAPI was used to mark the nuclei. Three separate experiments were shown by the photographs. Scale bar = 20 μm.

GO analysis of ZNF22 and HDAC family in glioma using bioinformatics revealed that ZNF22 and HDAC3 were involved in regulating transcription GO: 0006357 (*P* < 0.05) (Figure 1A). We downloaded the data through the GDC database (see text footnote 4) and analyzed it by bioinformatics statistics. We revealed ZNF22 (*P* < 0.05) (Figure 1B) and HDAC3 (*P* < 0.05) (Figure 1C) to be highly expressed in gliomas in a difference analysis. And according to survival curves, elevated HDAC3 expression was found to be detrimental to 5-year survival in gliomas (*P* < 0.05) (Figure 1D). Consequently, we hypothesized that the expression of ZNF22 and HDAC3 was enhanced in GECs, and that the high expression of ZNF22 and HDAC3 may have contributed to making BTB more permeable than BBB. QRT-PCR revealed that the relative expression of ZNF22 in GECs was 2,3-fold more than in ECs (Figure 2A). Western blot results (Figure 2B) presented that the expression of ZNF22 was significantly higher in GECs than that in ECs (*P* < 0.01). In GECs, ZNF22 was silenced by RNAi technology. *In vitro* BTB permeability was evaluated by measuring TEER and HRP flow. Figures 2C,D shows the findings, as contrast d to the ZNF22(–) NC group, the TEER values in the ZNF22(–) group were significantly higher (*P* < 0.001) and the HRP flux were significantly lower (*P* < 0.001). Neither TEER readings nor HRP flow were substantially different between the ZNF22(–) NC group and the control group.

Tight junction is a key functional component for preserving the integrity of the BBB and BTB, and variations in TJ-associated proteins are typically assumed to impact EC permeability. The expression of ZO-1, Occludin, and Claudin-5 was considerably increased in the ZNF22(–) group as contrasted to the ZNF22(–) NC group, as shown by Western blotting (Figure 2E). The control group and the ZNF22(–) NC group did not vary from one another in a way that was statistically significant (Figure 2E). The expression and distribution of TJ-associated proteins were further examined by applying immunofluorescence, and the changes in the expression of ZO-1, Occludin, and Claudin-5 shown in Figure 2F were comparable with the findings of Western blot. ZO-1, Occludin, and Claudin-5 became more linear in the ZNF22(–) group GECs as contrasted to the control and ZNF22(–) NC groups, as shown by immunofluorescence staining.

### ZNF22 as a transcription factor for ZO-1, Occludin, and Claudin-5 repressed their expression by binding to their promoter regions

The transcription factor ZNF22 controls the expression by binding specifically to the promoter region of target genes. The JASPAR database was applied to predict the binding sites of ZNF22 in the promoter regions of ZO-1, Occludin, and Claudin-5, and the outcomes demonstrated the promoter sequences of all three of the above each contained one potential ZNF22 binding site.

**FIGURE 3:**
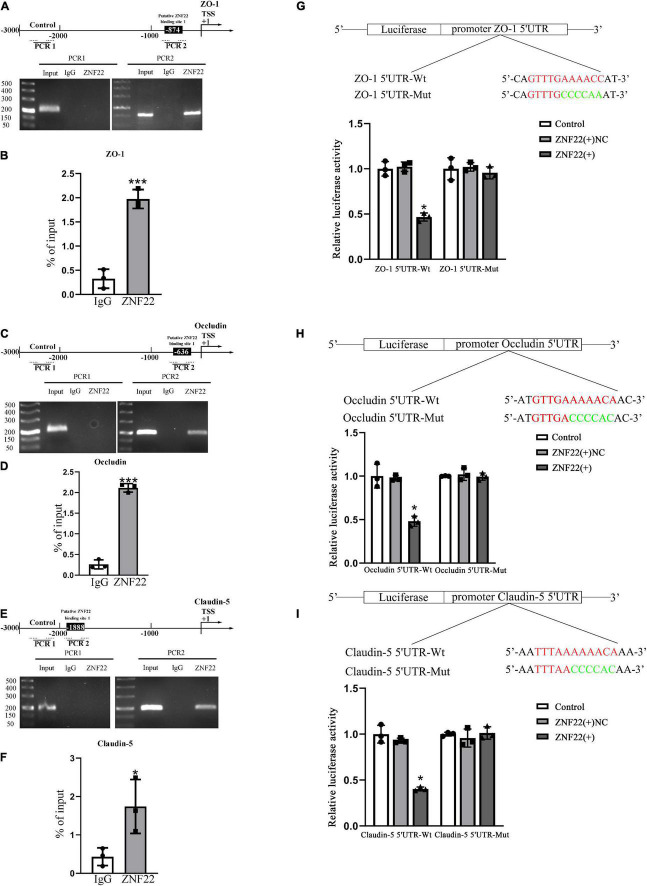
In the GECs of the BTB, ZNF22 binds to the promoters of TJ-associated proteins. **(A)** The human ZO-1 promoter regions are shown schematically at 3,000 bp upstream of the transcription start site (marked as TSS, + 1). Using their unique primers, ChIP PCR results for binding sites and upstream areas not anticipated to connect with ZNF22 were amplified by PCR. The Images served as examples of separate ChIP studies. **(B)** Results of three independent ChIP-qPCRs to confirm the presence of ZNF22 binding to specific regions of the promoter of ZO-1. ***p < 0.001 vs. IgG group. **(C)** The human Occludin promoter regions are shown schematically in 3,000 bp increments upstream of the transcription start site (TSS, denoted as + 1). Using specified primers, ChIP PCR results for binding locations and upstream areas not anticipated to connect with ZNF22 were amplified. The images were taken from independent ChIP tests. **(D)** Results of three independent ChIP-qPCRs to confirm the presence of ZNF22 binding to specific regions of the promoter of Occludin. ***p < 0.001 vs. IgG group. **(E)** The human Claudin-5 promoter regions are shown schematically in 3,000 bp increments upstream of the transcription start site (TSS, denoted as + 1). Using specified primers, ChIP PCR results for binding locations and upstream areas not anticipated to connect with ZNF22 were amplified. The images were taken from independent ChIP tests. **(F)** Results of three independent ChIP-qPCRs to confirm the presence of ZNF22 binding to specific regions of the promoter of Claudin-5. *p < 0.05 vs. IgG group. **(G)** ZNF22 and ZO-1 binding sites in GECs were identified using dual luciferase reporter assays. Data represent mean ± SD (n = 3, each). *p < 0.05 vs. ZNF22(+) NC group. ZNF22(+) NC group. **(H)** The binding sites of ZNF22 and Occludin in GECs were determined using dual luciferase reporter assays. Data represent mean ± SD (*n* = 3, each). *p < 0.05 vs. ZNF22(+) NC group. **(I)** ZNF22 and Claudin-5 binding sites in GECs were determined using dual luciferase reporter assays. Data represent mean ± SD (*n* = 3, each). *p < 0.05 vs.

Polymerase chain reaction primers and negative control primers containing the predicted sites were randomly designed in the 3000 bp promoter region. Their negative control regions did not show recruitment of ZNF22. In addition, ZNF22 was recruited to the ZO-1, Occludin, and Claudin-5 binding site in the predicted binding sites (Figures 3A,C,E). And the results of ChIP-qPCR showed the same conclusion (Figures 3B,D,F).

Next, we performed a dual luciferase reporter test to validate the possible binding locations of ZNF22 in the 5′ untranslated regions of ZO-1, Occludin, and Claudin-5. As shown in Figures 3G–I, the luciferase activity of the 5′UTR-Wt + ZNF22 (+) groups in ZO-1, Occludin, and Claudin-5 was lower than that of the 5′-UTR-Wt + ZNF22 (+) NC group in ZO-1, Occludin, and Claudin-5. However, the luciferase activities of GECs were maintained at the same level after co-transfection of reporter vectors containing the corresponding mutated ZO-1, Occludin, and Claudin-5 binding fragments with ZNF22 (+). The aforementioned studies demonstrated that ZNF22 suppressed transcription of ZO-1, Occludin, and Claudin-5 by targeting the aforementioned 5′-UTR regions of all three genes. These data demonstrated that ZNF22 binds to the promoter areas of ZO-1, Occludin, and Claudin-5 and inhibits their expression.

### HDAC3 expression was up-regulated in glioma-exposed endothelial cells and promoted blood tumor barrier permeability

**FIGURE 4:**
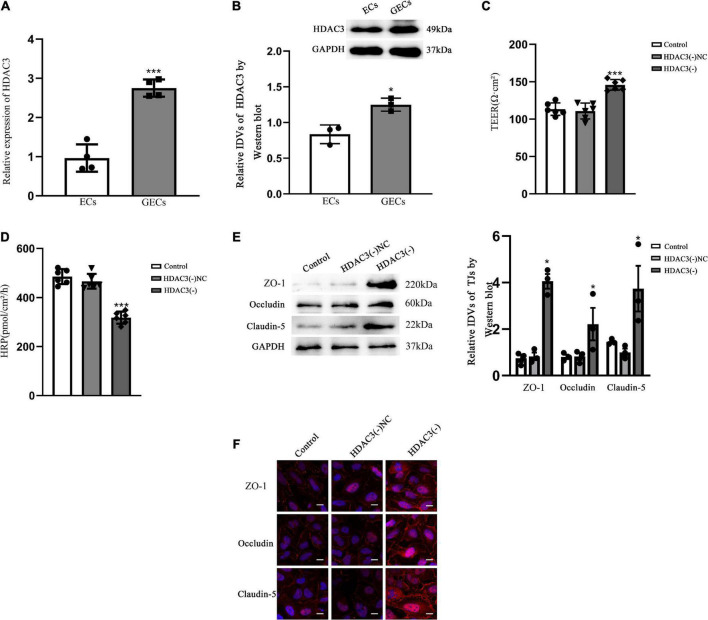
BTB GECs exhibited endogenous HDAC3 expression and were implicated in the control of the permeability of BTB *in vitro* **(A)** QRT-PCR was applied for determine the relative expression of HDAC3 in ECs and GECs. As an endogenous control, GAPDH protein levels were measured. Data represent mean ± SD (*n* = 3, each). ***p < 0.001 vs. ECs group. **(B)** Immunoblotting was used to detect HDAC3 protein expression. As an endogenous control, GAPDH protein levels were measured. Data represent mean ± SD (*n* = 3, each). *p < 0.05 vs. ECs group. **(C)** TEER values of GECs are expressed as Ωcm^2^. Data are expressed as mean ± SD (*n* = 3, each group). ***p < 0.001 vs. HDAC3(–) NC group. **(D)** HRP flow was calculated as pmol/cm^2^/h. Data are expressed as mean ± SD (*n* = 3, each). ***p < 0.001 vs. HDAC3(–) NC group. **(E)** Protein blot analysis of TJ-associated proteins ZO-1, Occludin, and Claudin-5 in GECs. As an endogenous control, GAPDH protein levels were measured. Data are expressed as mean ± SD (*n* = 3, each). *p < 0.05 vs. HDAC3(–) NC group. **(F)** Immunofluorescence localization of ZO-1, Occludin, and Claudin-5 in GECs. The nuclei were marked with DAPI, and fluorescent secondary antibodies were used to mark ZO-1 (red), Occludin (red), and Claudin-5 (red). The pictures showed what happened in three different experiments. Scale bar = 20 μm.

qRT-PCR demonstrated that the relative expression of HDAC3 was 2.5-fold higher in GECs than in ECs (*P* < 0.001) (Figure 4A). Western blot results (Figure 4B) presented that the expression of HDAC3 was significantly higher in GECs than that in ECs (*P* < 0.05). The permeability of BTB was measured by using TEER values and HRP flux. TEER values were significantly elevated (*P* < 0.001) and HRP flux were significantly restrained as contrasted to HDAC3 (–) NC groups (*P* < 0.001). Neither TEER readings nor HRP flux differed significantly between the control and HDAC3 (–) NC groups (Figures 4C,D).

The HDAC3(–) group markedly expanded the expression of ZO-1, Occludin, and Claudin-5 contrasted to the HDAC3(–) NC group, as shown by Western blotting (*P* < 0.05). There were no statistical significance between the control group and HDAC3(–) NC (Figure 4E). The expression and distribution of TJ-associated proteins were further studied by immunofluorescence, and the alterations in the expression of ZO-1, Occludin, and Claudin-5 that can be observed in Figure 4F were similar with the results shown in Figure 4E. In the HDAC3(–) group, ZO-1, Occludin, and Claudin-5 became more linear than in the HDAC3(–) NC group, as shown by immunofluorescence staining.

### ZNF22 interacted with HDAC3 to block the transcriptional activity of the target gene TJ-associated proteins, which led to activating blood tumor barrier permeability

**FIGURE 5:**
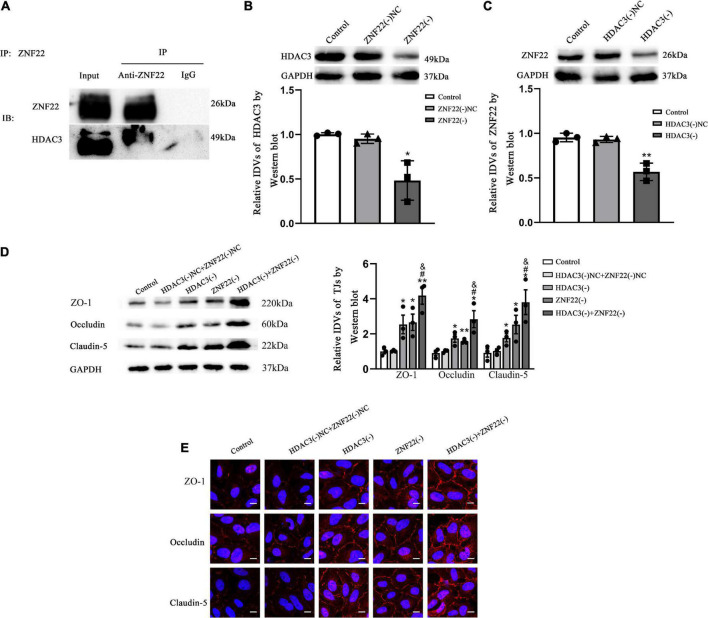
ZNF22 interacted with HDAC3 to block the expression of TJ-associated proteins, which led to activating BTB permeability. **(A)** Interaction between endogenous ZNF22 and HDAC3 was detected by immunoprecipitation in GECs. **(B)** Protein blot analysis of HDAC3 expression in GECs. As an endogenous control, GAPDH protein levels were measured. Data were expressed as mean ± SD (*n* = 3, each). *p < 0.05 vs. ZNF22(–) NC group. **(C)** Protein blot analysis of ZNF22 expression in GECs. As an endogenous control, GAPDH protein levels were measured. Data represent mean ± SD (*n* = 3, each). **p < 0.01 vs. ZNF22(–) NC group. **(D)** In GECs, protein blot analysis revealed the expression of TJ-associated proteins ZO-1, Occludin, and Claudin-5. As an endogenous control, GAPDH protein levels were measured. The data is presented as mean ± SD (*n* = 3, each). *p < 0.05 and **p < 0.01 vs. HDAC3(–) NC + ZNF22(–) NC group. ^#^*p* < 0.05 vs. HDAC3(–) group. ^&^*p* < 0.05 vs. ZNF22(–) group. **(E)** ZO-1, Occludin, and Claudin-5 immunofluorescence localization in GECs. Fluorescent secondary antibodies were used to label ZO-1 (red), Occludin (red), and Claudin-5 (red), while DAPI was used to label nuclei. The photos were taken from three separate trials. Scale bar = 20 μm.

To elucidate the molecular mechanism of ZNF22 in affecting BTB permeability, we next identified the interaction between ZNF22 and HDAC3 in GECs by means of Co-IP assay (Figure 5A). The results showed that ZNF22 and HDAC3 have protein interaction relationship in GECs.

Interestingly, after knockdown of ZNF22 in GECs, the expression of HDAC3 was significantly reduced (*P* < 0.05) in the ZNF22(–) group as contrasted to the ZNF22(–) NC group (Figure 5B). Similarly, after knockdown of HDAC3 in GECs, ZNF22 expression was significantly hindered (*P* < 0.01) in the HDAC3(–) group as contrasted to the HDAC3(–) NC group (Figure 5C).

Western-blot results demonstrated that the expression of ZO-1, Occludin, and Claudin-5 in the HDAC3(–) group and ZNF22(–) group was enhanced contrasted to the HDAC3(–) NC and ZNF22(–) NC group, respectively, (*P* < 0.05). The expression of ZO-1, Occludin, and Claudin-5 in the HDAC3(–) + ZNF22(–) group was significantly increased contrasted to the HDAC3(–) group and ZNF22(–) group (*P* < 0.05). The differences among the control, HDAC3(–) NC and ZNF22(–) NC groups were not statistically significant (Figure 5D).

**FIGURE 6:**
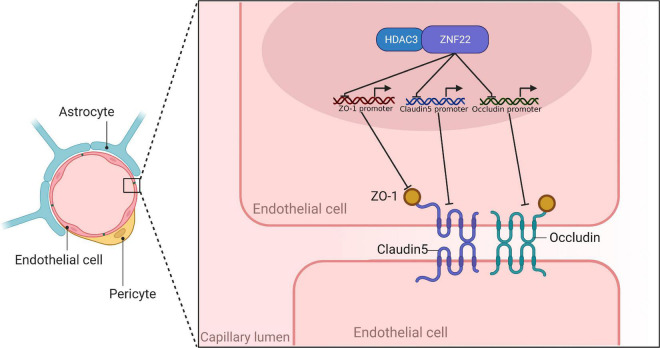
Schematic diagram of ZNF22 and HDAC3 to regulate BTB permeability.

ZO-1, Occludin, and Claudin-5 became more linear in the HDAC3(–) and ZNF22(–) groups against HDAC3(–) NC and ZNF22(–) NC groups, as shown by immunofluorescence staining. ZO-1, Occludin, and Claudin-5 became more linear in the HDAC3(–) + ZNF22(–) group contrasted to the HDAC3(–) and ZNF22(–) groups (Figure 5E). In addition, a schematic illustration of ZNF22 and HDAC3 modulation of BTB permeability was presented in Figure 6.

## Discussion

In the current research, we discovered for the first time that ZNF22 expression was upregulated in GECs, and ZNF22 as a transcriptional repressor bound to the promoter region of TJ-associated proteins to inhibit their expression, which led to an increase in BTB permeability. Meanwhile, HDAC3, as an assisted transcription factor of ZNF22, mediated BTB permeability regulation through its interaction with ZNF22. We provided direct evidence for the involvement of ZNF22 in the regulation of BTB permeability. To our knowledge, this was the first report to utilize ZNF22 as a transcription factor to modulate the mechanism of BTB permeability.

At present, there is still no effective cure for glioma, and the search for a cure is ongoing. The existence of BBB inhibits about one hundred percent of big molecules and more than ninety-eight percent of tiny molecules from entering (Jiang, 2013). For this reason, various strategies have been adopted to bypass the BBB, such as invasive intracerebral administration or non-invasive nasal administration (Chen and Liu, 2012), or transient BBB destruction caused by biological, chemical or physical stimuli (e.g., occluded small band toxins, mannitol, magnetic heating and ultrasound), which in turn delivers the drug *via* the circulatory system to the brain *via* the blood (Neuwelt et al., 1991). Tight junction proteins almost completely block the passage of hydrophilic molecules through the paracellular gaps. And increasing the permeability of the BBB by regulating TJ-associated proteins to provide a new route for drug penetration is one of the ideas for the treatment of glioma.

Same as CNS tumors, most of the alternative drugs for CNS infections cannot be used in the clinic because they do not cross the BBB at a sufficient level to have a therapeutic effect (Miller et al., 2008). Thus, again, the most important factor limiting the development of new drugs for the CNS is the BBB (Pardridge, 2007). Increasing the permeability of the BBB to drugs may provide additional therapeutic tools for CNS infections.

Numerous studies have revealed transcription factors that play a crucial role in regulating BBB permeability. Zinc finger proteins (ZNF) were a large class of transcription factors with multiple functions that played an important role in tumorigenesis and progression. The transcription factor ZNF345C acted as a transcriptional repressor, inhibiting endothelial angiogenic sprouting through the KRAB structural domain (Oo et al., 2020). The transcription factor ZNF655 promoted glioma progression by binding to the promoter of AURKA (Chen et al., 2022). In previous studies, ZNF22 was thought to be involved in the development of teeth (Gao et al., 2003), and its mechanism of action in gliomas or others was still not thoroughly elucidated. The transmembrane proteins Claudins and Occludin have been investigated the most among the known compounds related with TJ. Claudin-5 was shown to be involved in the size-selective loosening of the permeability of the mouse BBB, which influenced the permeability of molecules smaller than 800 Da (Nitta et al., 2003). And Occludin was shown to influenced BBB permeability by composing TJ-strand branching points and regulating their complexity (Saito et al., 2021). ZO-1 was the first protein to be shown to be positively correlated with TJ (Stevenson et al., 1986), it linked the transmembrane protein of TJ to the actin cytoskeleton (Fanning et al., 1998), this interaction may be essential for the stability and function of the TJ. It also co-localized with transcription factors (Balda and Matter, 2000) and G-proteins (Meyer et al., 2002). Consequently, the regulation of these three proteins by ZNF22 would have a sweeping and multifaceted effect on the BBB/BTB permeability. This may provide a new direction for future research into the role of ZNF22 in glioma.

HDACs were classified into four classes based on their function and sequence homology (de Ruijter et al., 2003). Among them, HDAC3 and HDAC11 were observed to be highly expressed in the rat brain (Broide et al., 2007). HDAC inhibitors were demonstrated to increase epithelial barrier function by oligomerizing the TJ protein ZO-3, Occludin and CGN in the cell membrane (Nakano et al., 2022). A study reported that HDAC1 promoted the permeability of the BBB by inhibiting the expression of Claudin-5 (Dudek et al., 2020). Prior research centered on the relationship between HDAC3 and glioma development. Nonetheless, the precise link between HDAC3 expression and glioma patient prognosis remains contested. Some studies showed that a high expression of HDAC3 predicted a good prognosis, while others showed the opposite result. In the present experiment, we found that HDAC3 played a positive role in the treatment of gliomas by increasing the permeability of the BBB/BTB. Similar to our results, inhibition of HDAC3 in a mouse model of type 2 diabetes enhanced BBB permeability through activated Nrf2 (Zhao et al., 2019). Similarly, another experiment showed that MiR-193b-3p reduced the permeability of the BBB by inhibited HDAC3 expression and activity (Lai et al., 2020). These experiments suggested that HDAC3 affected the permeability of the BBB/BTB through various pathways.

Protein interactions play a fundamental role in transcriptional regulation. In our present study, the results presented that ZNF22 interacting with the HDAC3 inhibited the expression of TJ-associated proteins. Similarly, some studies have found that the interaction of co-repressors CtBP1 and HDAC3 with Glis2 was involved in the repression of transcription by Glis2 (Kim et al., 2005). The interaction between HDAC3 and KLF6 was identified as a potential mechanism for adipogenesis, with KLF6 as a transcriptional regulator to inhibit the mediator of adipocyte differentiation (Li et al., 2005). HDAC3 interacted directly with tissue-specific transcription factors to repress the transcription, such as binding to GATA-2 to inhibit the GATA-2-dependent target genes (Ozawa et al., 2001). In addition to its effects through its intrinsic deacetylase activity, HDAC3 established direct protein-protein interactions and exerted its transcriptional co-repression function (Gallinari et al., 2007).

In summary, our study showed that in GECs, elevated ZNF22, a transcription factor for TJ-associated proteins, repressed the expression of ZO-1, Occludin, and Claudin-5, while the protein interaction of HDAC3 with ZNF22 enhanced the transcriptional repression of ZNF22 and ultimately increased the permeability of BTB. These studies may provide new therapeutic strategies for CNS tumors and infections.

## Data availability statement

The datasets presented in this study can be found in online repositories. The names of the repository/repositories and accession number(s) can be found in the article/Supplementary material.

## Author contributions

BZ and TM: conceptualization. BZ, LZ, and HN: data curation and investigation. BZ and LZ: formal analysis, software, validation, and visualization. XZ and TM: funding acquisition, project administration, and resources. BZ, HN, and TM: methodology. TM: supervision and writing—review and editing. BZ: writing—original draft. All authors contributed to the article and approved the submitted version.

## Footnotes

1
https://jaspar.genereg.net/

2
http://genemania.org/

3
https://cn.string-db.org/

4
https://portal.gdc.cancer.gov/
