# Supplementary material for: Transcription factor ZNF22 regulates blood-tumor barrier permeability by interacting with HDAC3 protein
Source: Front Mol Neurosci. 2022 Nov 28;15:1027942. doi: 10.3389/fnmol.2022.1027942 (PMC9742255; doi:10.3389/fnmol.2022.1027942)
Supplement: Supplementary file 1 [file Table_1.DOCX]

A

The primers of qRT-PCR.

| ZNF22-R | 5ʹ-gggagaaaccctaccagtgc-3ʹ |
| --- | --- |
| ZNF22-F | 5ʹ-ccgggttttacgaggcttct-3ʹ |
| HDAC3-F | 5ʹ-cagattcatgacgtgcctgc-3ʹ |
| HDAC3-R | 5ʹ-cctccccagcaagcctattg-3ʹ |
| Occludin-F | 5ʹ-tgtgggataaggaacacatttatga-3ʹ |
| Occludin-R | 5ʹ-cagacacatttttaaccccatcttca-3ʹ |
| Claudin-5-F | 5ʹ-tctgctggttcgccaacat-3ʹ |
| Claudin-5-R | 5ʹ-cggcaccgtcggatca-3ʹ |
| ZO-1-F | 5ʹ-tgaacgctctcataagcttcgtaa-3ʹ |
| ZO-1-R | 5ʹ-accgtaccaaccatcattcattg-3ʹ |
| GAPDH-F | 5ʹ-ccatggggaaggtgaaggtc-3ʹ |
| GAPDH-R | 5ʹ-gaaggggtcattgatggcaac-3ʹ |

B

The primers of Cell transfection

| sh-ZNF22 :5ʹ-CTTGGAAAGCTGGTACAGGAA-3ʹ |
| --- |
| sh-HDAC3 :5ʹ- CCTTCCACAAATACGGAAATT-3ʹ |

C

The primers of ChIP

| Claudin-5-F | 5ʹ-TTTGAGACCAGCCTGCACAA-3ʹ |
| --- | --- |
| Claudin-5-R | 5ʹ-TTTGTCATCGAGGCTGCAGT-3ʹ |
| Occludin-R | 5ʹ-CGCTTTCAATGCAGATAGTTAAATG-3ʹ |
| Occludin-F | 5ʹ-TCTGGATTCTGTTTCCAAGGGA-3ʹ |
| ZO-1-F | 5ʹ-GCTAGGAAGGGATGTGGCAG-3ʹ |
| ZO-1-R | 5ʹ-CTACATGTAGGGCTGCTGGG-3ʹ |

D

Wild-type and mutant plasmid sequences

| ZO-1 5'UTR-Wt | 5ʹ-CAGTTTGAAAACCAT-3ʹ |
| --- | --- |
| ZO-1 5'UTR-Mut | 5ʹ-CAGTTTGCCCCAAAT-3ʹ |
| Occludin 5'UTR-Wt | 5ʹ-ATGTTGAAAAACAAC-3ʹ |
| Occludin 5'UTR-Mut | 5ʹ-ATGTTGACCCCACAC-3ʹ |
| Claudin-5 5'UTR-Wt | 5ʹ-AATTTAAAAAACAAA-3ʹ |
| Claudin-5 5'UTR-Mut | 5ʹ-AATTTAACCCCACAA-3ʹ |
