# Supplementary figures and images for: Transcription factor ZNF22 regulates blood-tumor barrier permeability by interacting with HDAC3 protein
Source: Front Mol Neurosci. 2022 Nov 28;15:1027942. doi: 10.3389/fnmol.2022.1027942 (PMC9742255; doi:10.3389/fnmol.2022.1027942)

A

Predicted results in GeneMAINA and String.


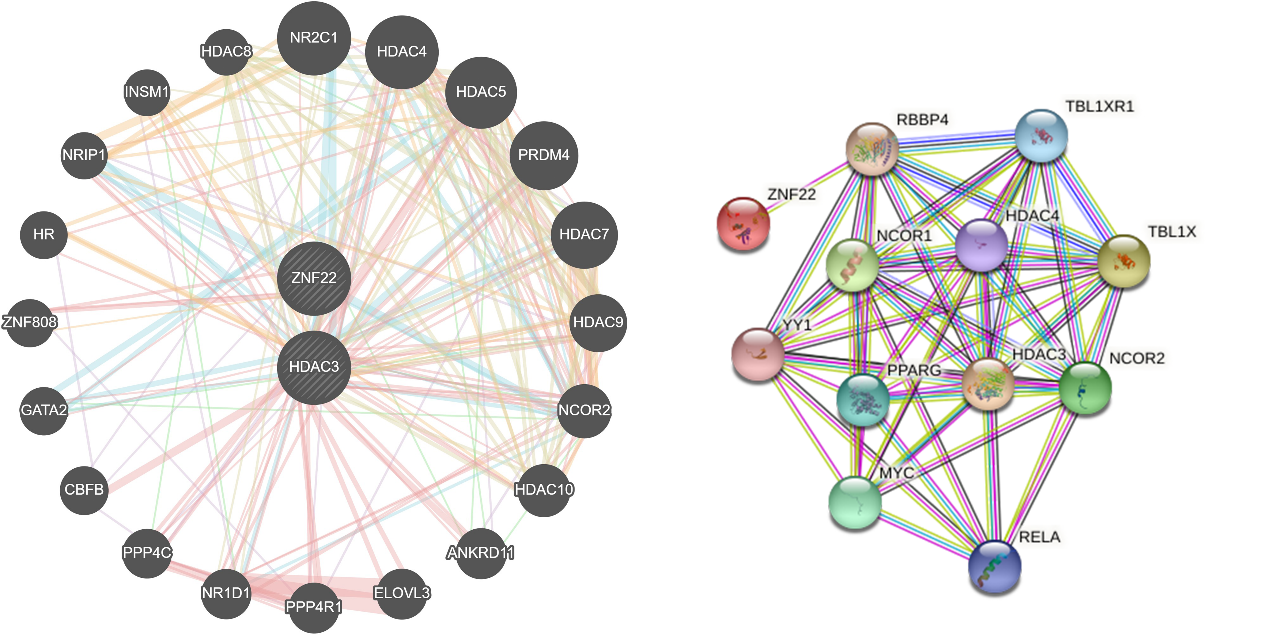

Supplement: Supplementary file 2 [file Table_2.DOCX]

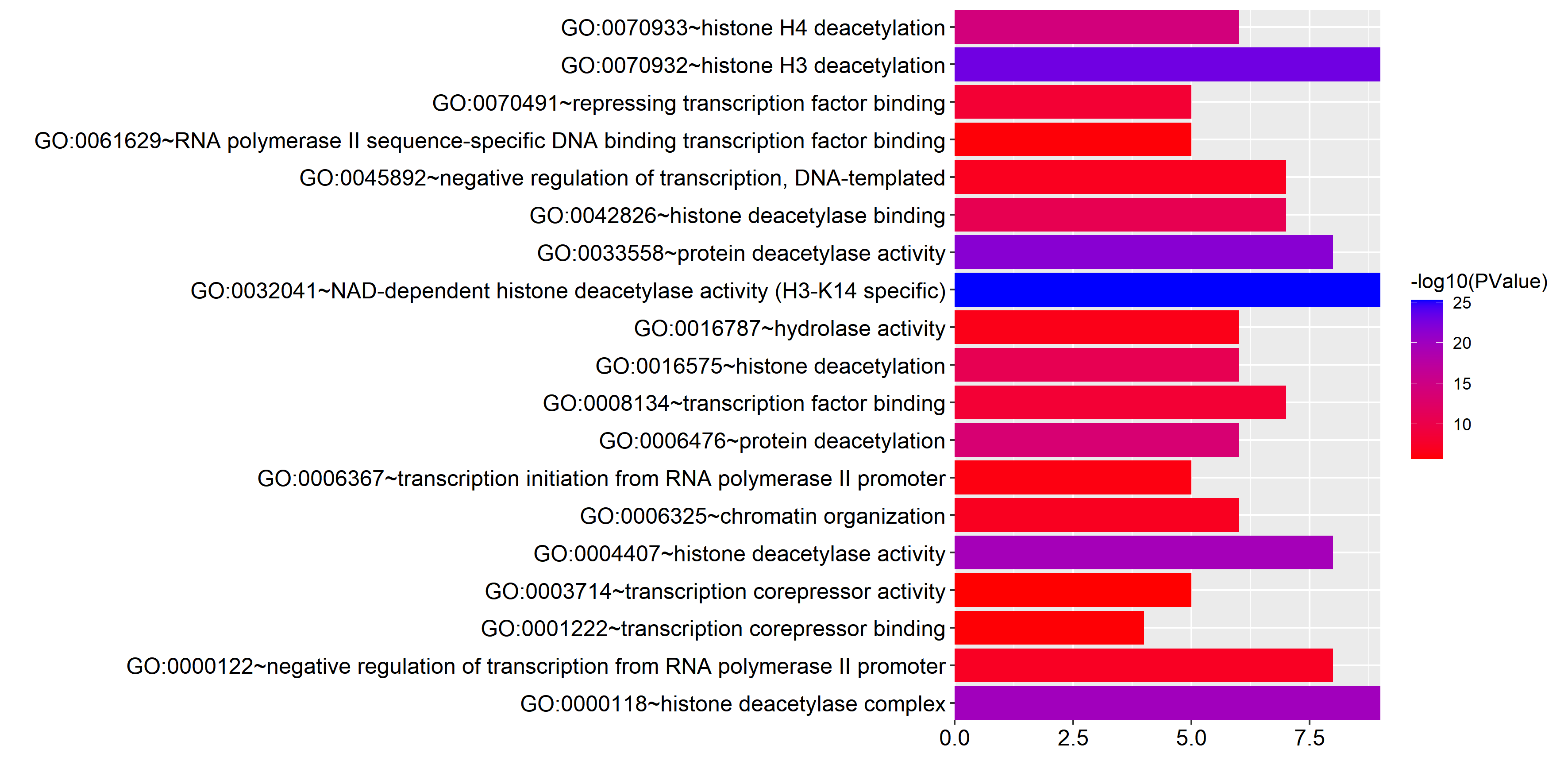

Supplement: Supplementary file 3 [file Data_Sheet_1.ZIP › go/goPvalue.tiff]

ZO-1 of shZNF22

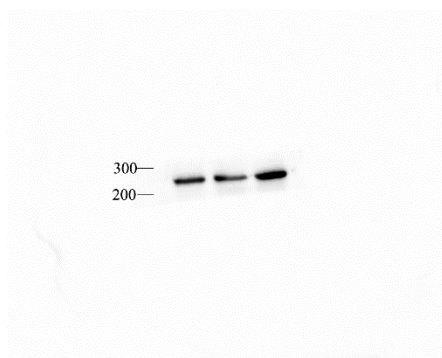

Occludin of shZNF22

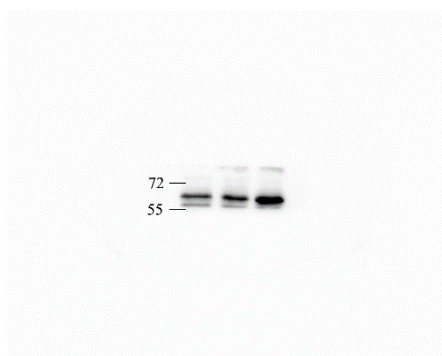

Claudin-5 of shZNF22

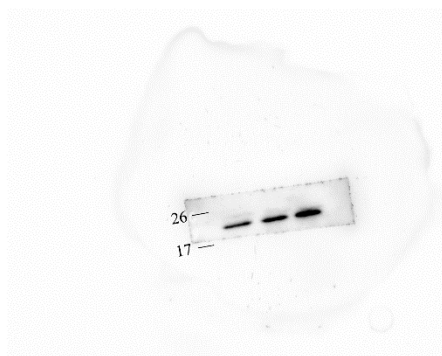

GAPDH of shZNF22

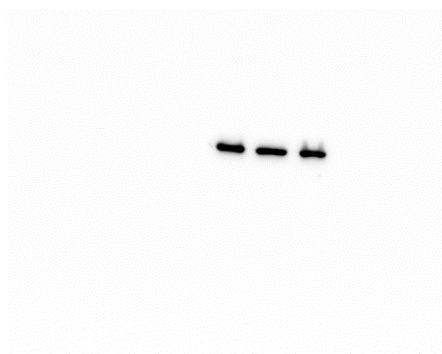

Supplement: Supplementary file 4 [file Data_Sheet_2.ZIP › FIG2 E/FIG2 E Original Western blot pictures.pdf]

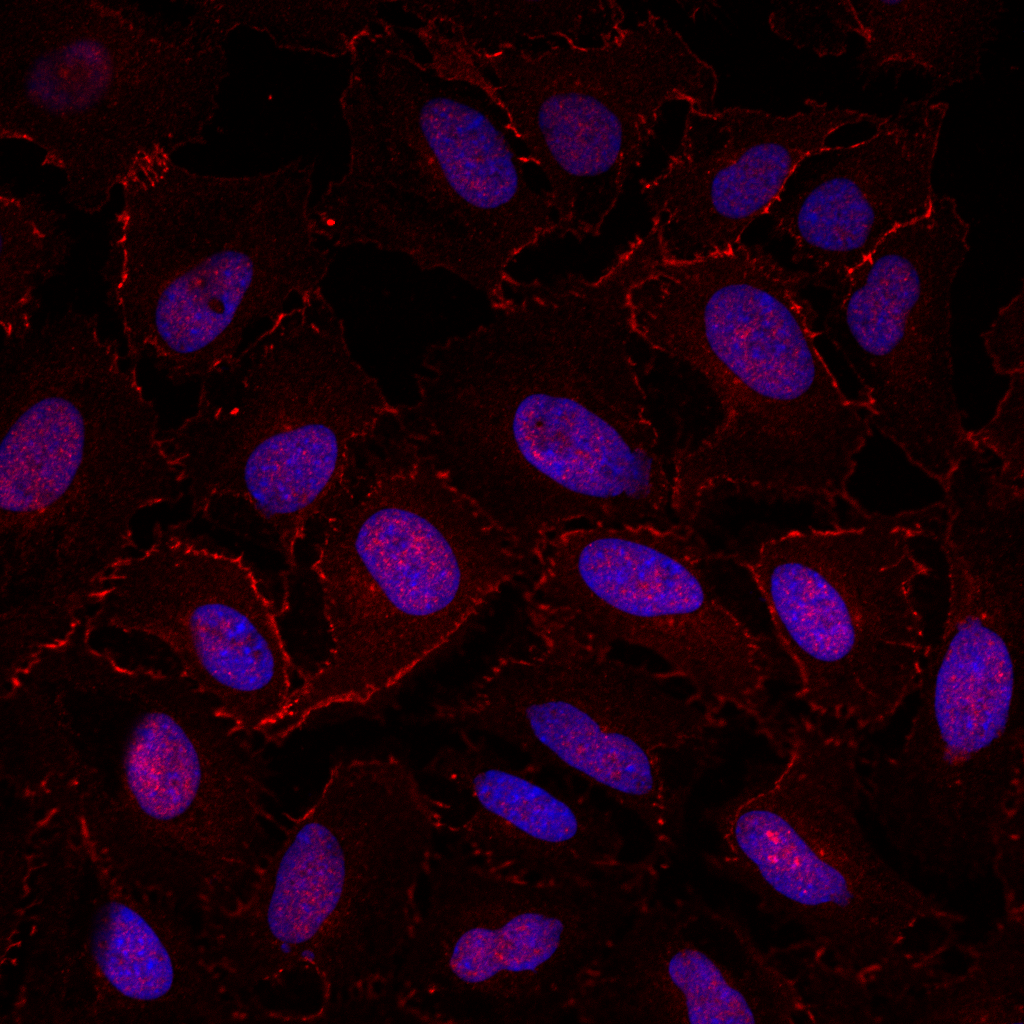

Supplement: Supplementary file 4 [file Data_Sheet_2.ZIP › FIG2 F/control/Claudin-5 control1.tif]

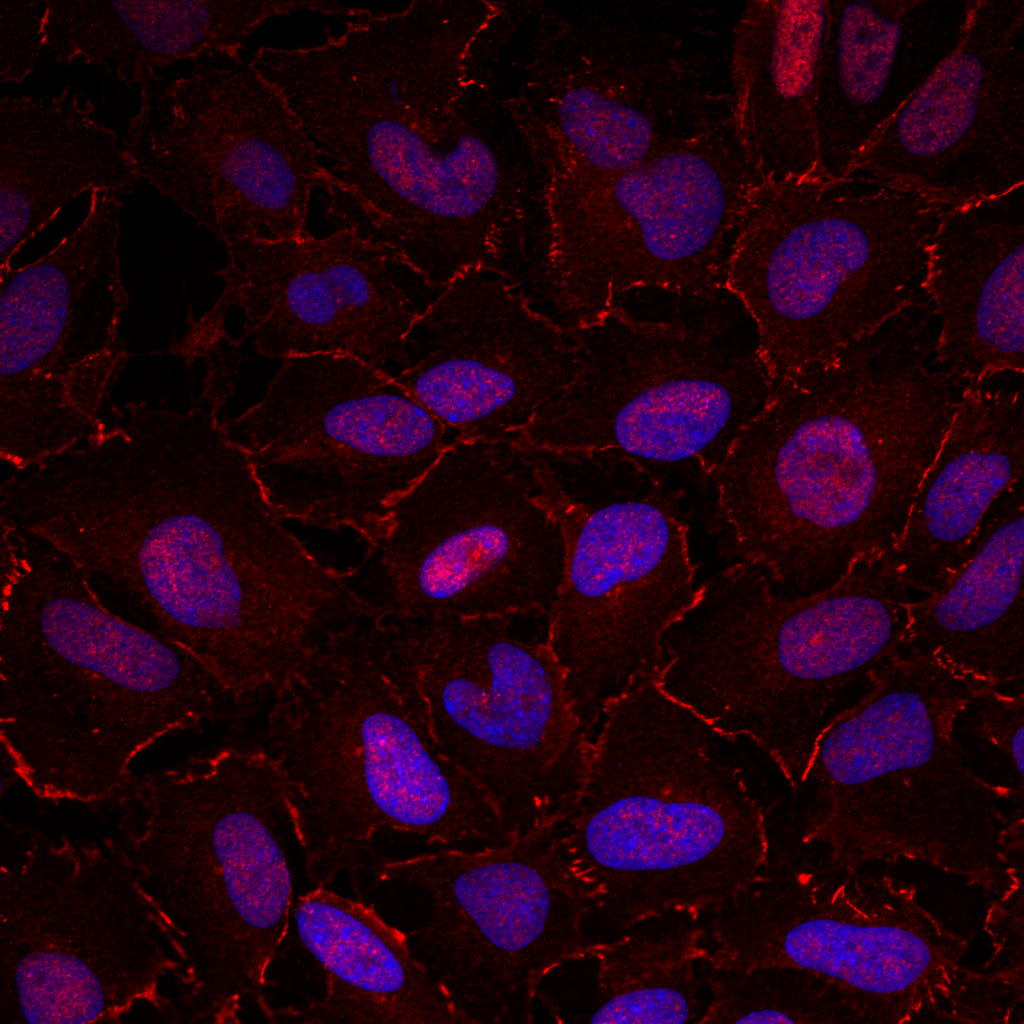

Supplement: Supplementary file 4 [file Data_Sheet_2.ZIP › FIG2 F/control/Occludin control 1.tif]

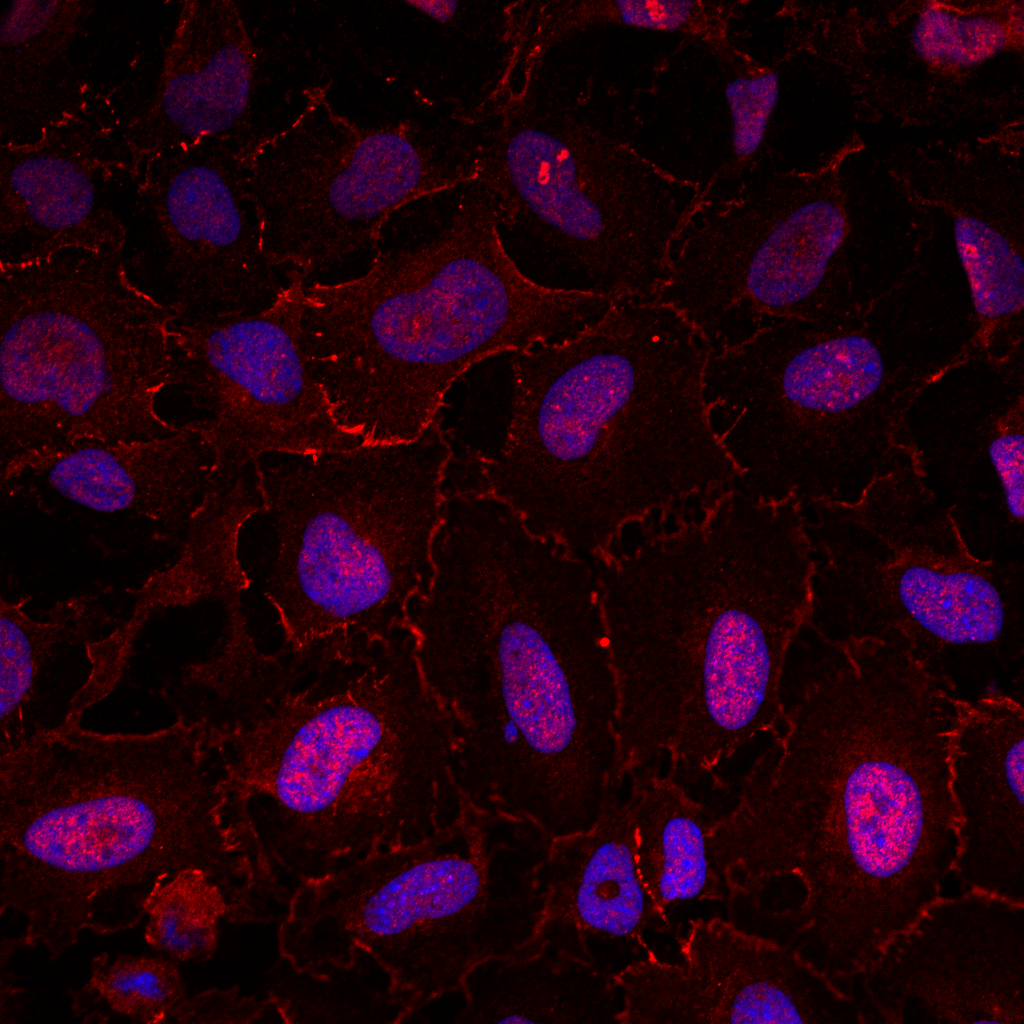

Supplement: Supplementary file 4 [file Data_Sheet_2.ZIP › FIG2 F/control/ZO-1 control 1.tif]

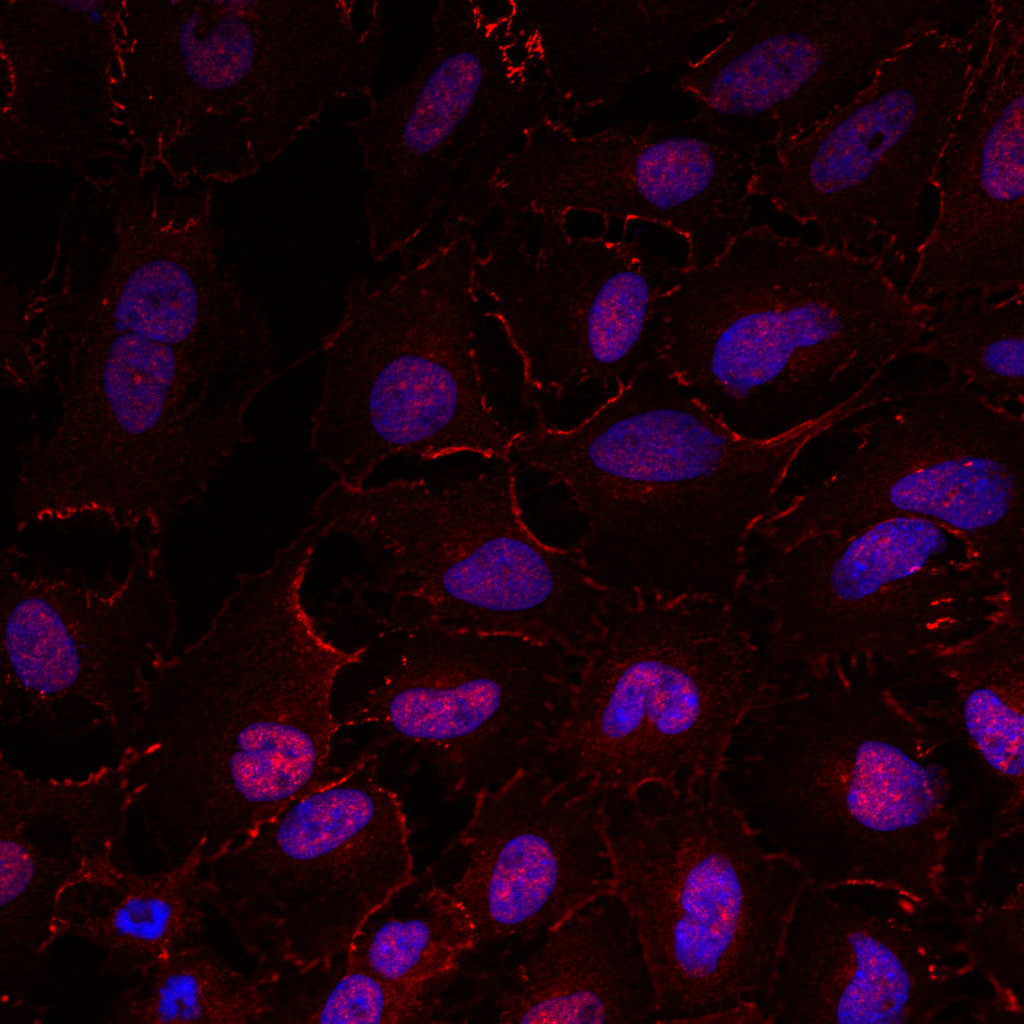

Supplement: Supplementary file 4 [file Data_Sheet_2.ZIP › FIG2 F/NC/CLAUDIN5 NC.tif]

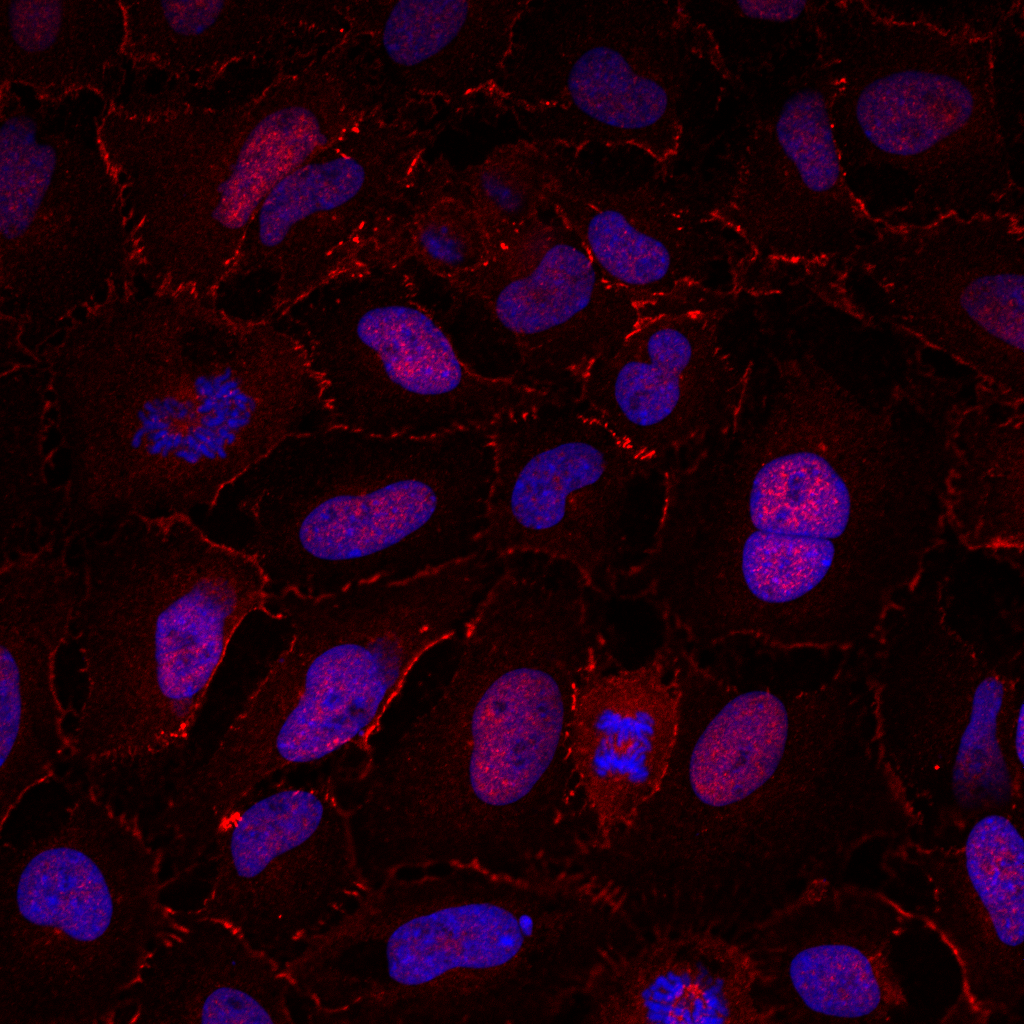

Supplement: Supplementary file 4 [file Data_Sheet_2.ZIP › FIG2 F/NC/OCCLUDIN NC.tif]

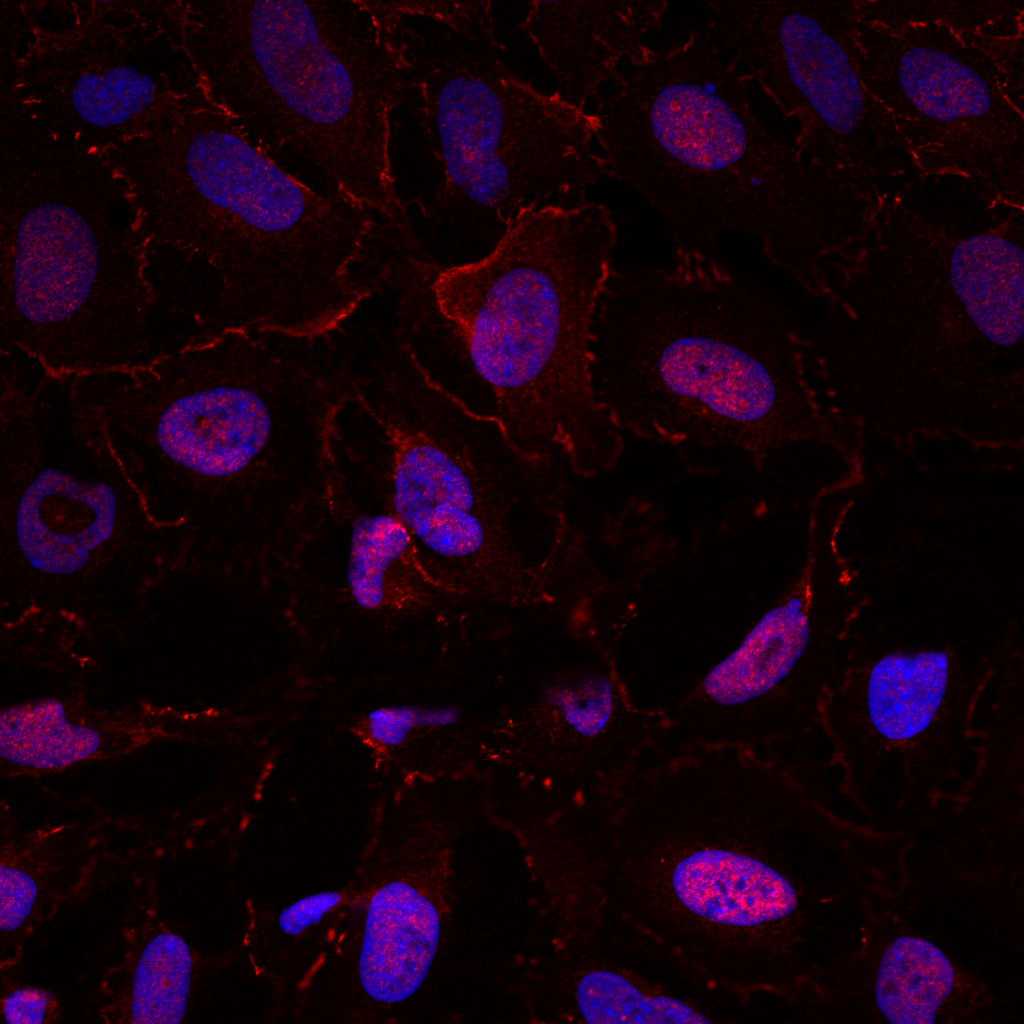

Supplement: Supplementary file 4 [file Data_Sheet_2.ZIP › FIG2 F/NC/zo-1 NC .tif]

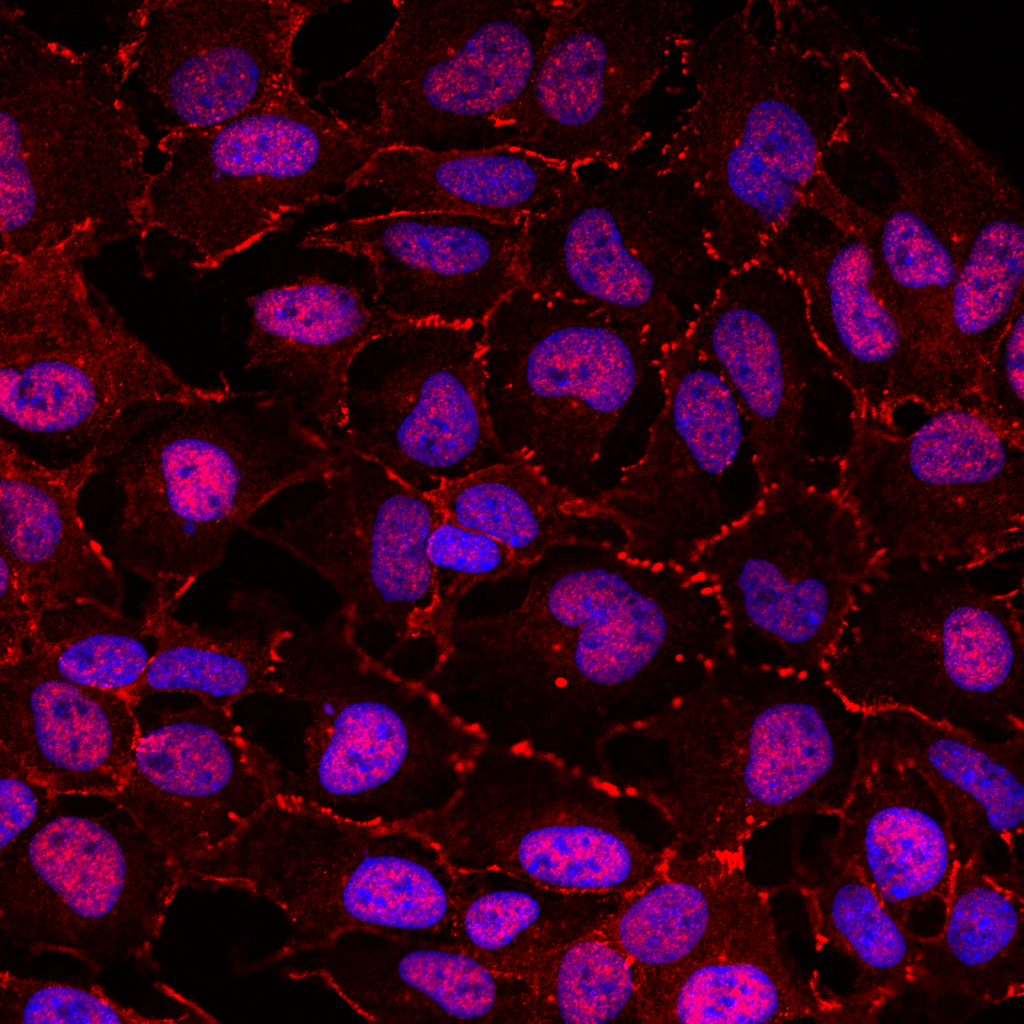

Supplement: Supplementary file 4 [file Data_Sheet_2.ZIP › FIG2 F/ZNF22(-)/CLUDIN5 ZNF22(-).tif]

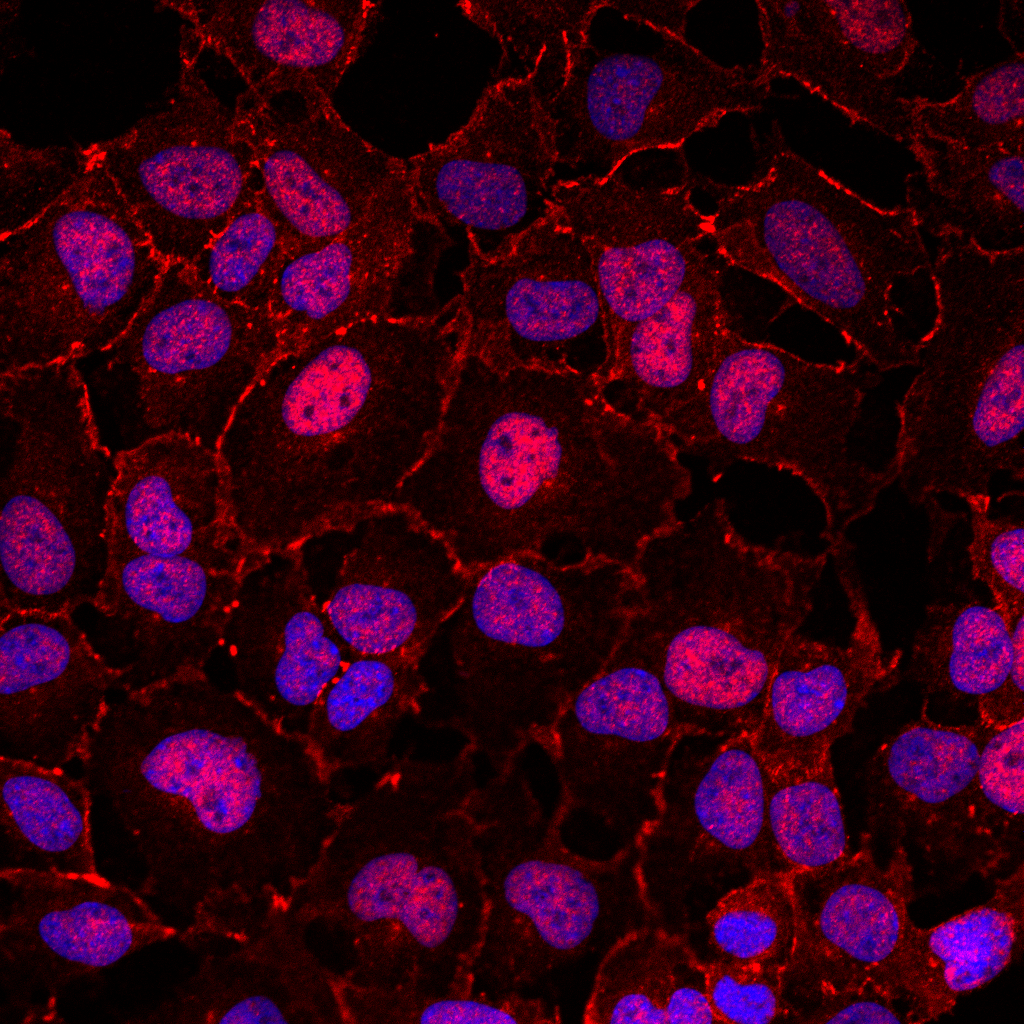

Supplement: Supplementary file 4 [file Data_Sheet_2.ZIP › FIG2 F/ZNF22(-)/OCCLUDIN ZNF22(-).tif]

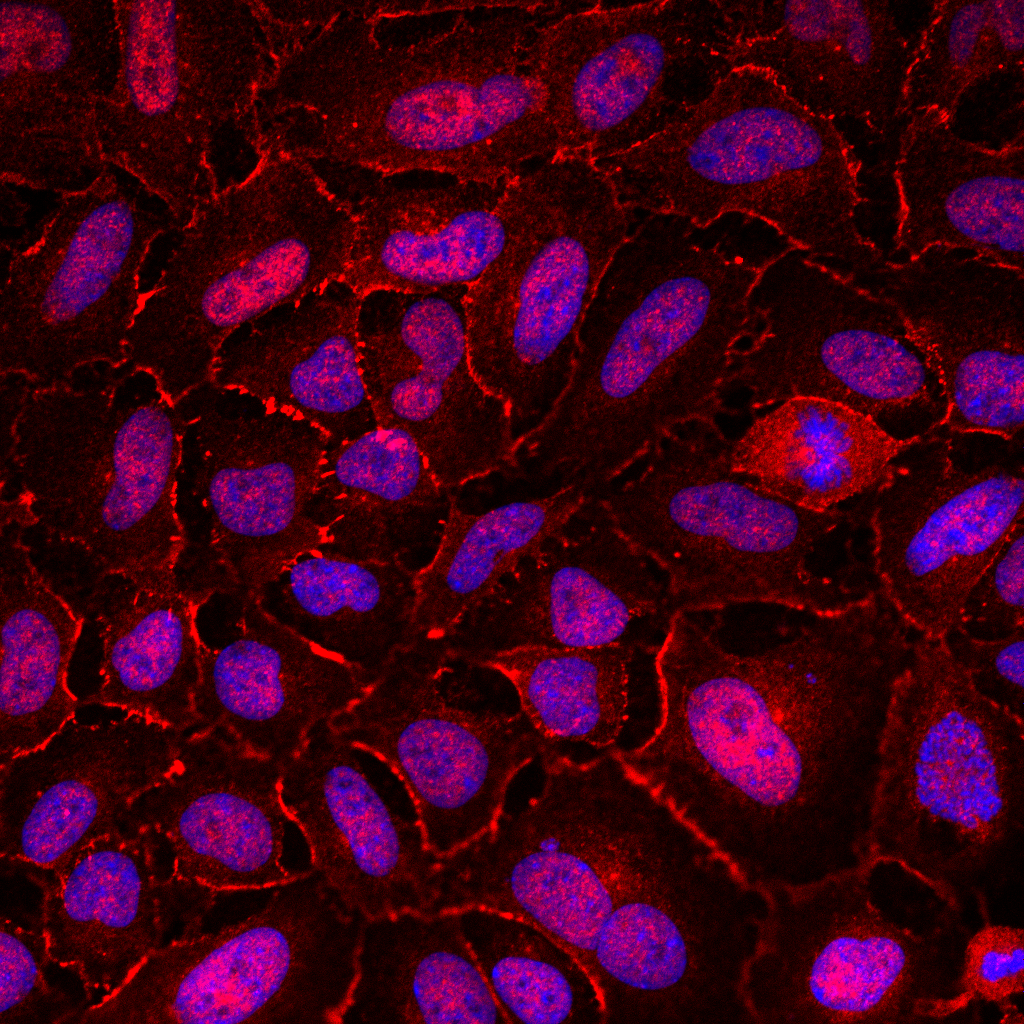

Supplement: Supplementary file 4 [file Data_Sheet_2.ZIP › FIG2 F/ZNF22(-)/zo-1 ZNF22(-).tif]

Relative expression of HDAC3

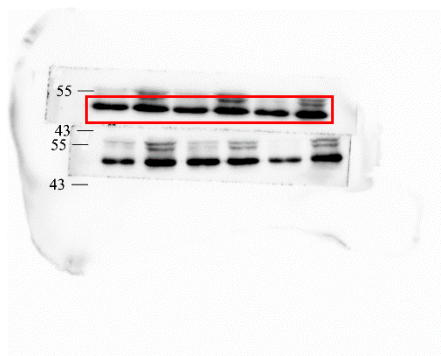

GAPDH of relative expression of HDAC3

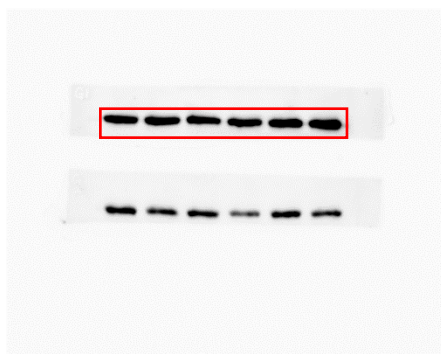

Supplement: Supplementary file 6 [file Data_Sheet_4.ZIP › FIG4 B/FIG4 B Original Western blot pictures .pdf]

FIG 5 E

ZO-1 of shHDAC3

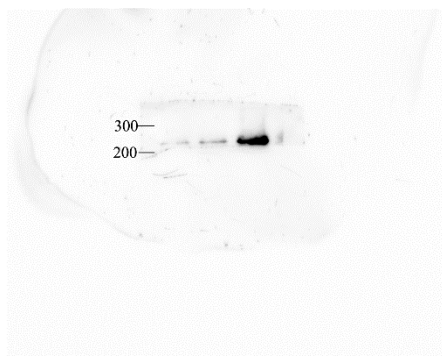

Occludin of shHDAC3

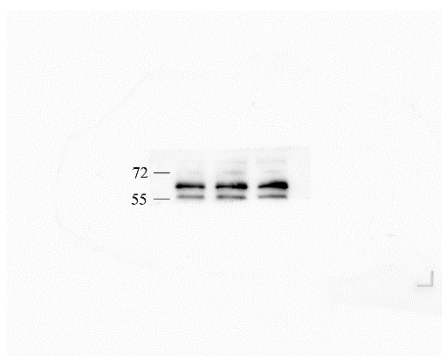

Claudin-5 of shHDAC3

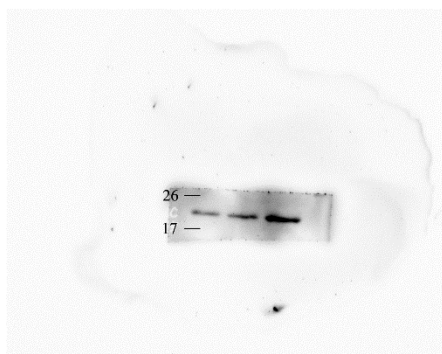

GAPDH of shHDAC3

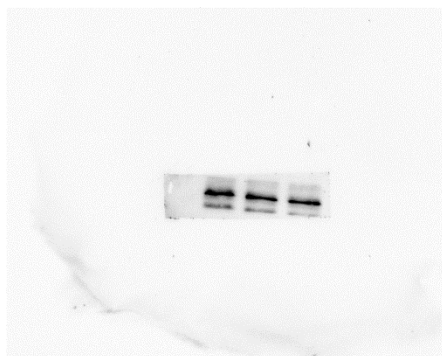

Supplement: Supplementary file 6 [file Data_Sheet_4.ZIP › FIG4 E/FIG4 E Original Western blot pictures.pdf]

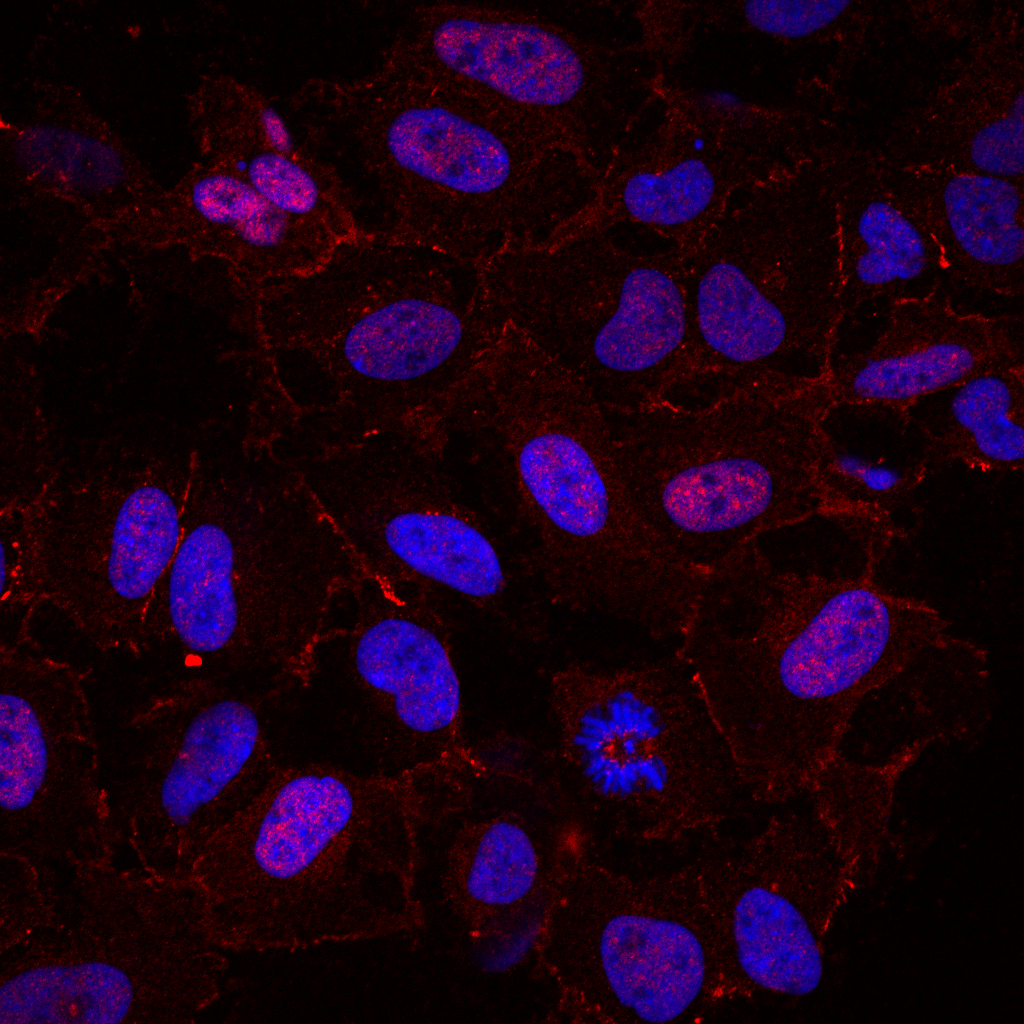

Supplement: Supplementary file 6 [file Data_Sheet_4.ZIP › FIG4 F/control/CLAUDIN5 CONTROL.tif]

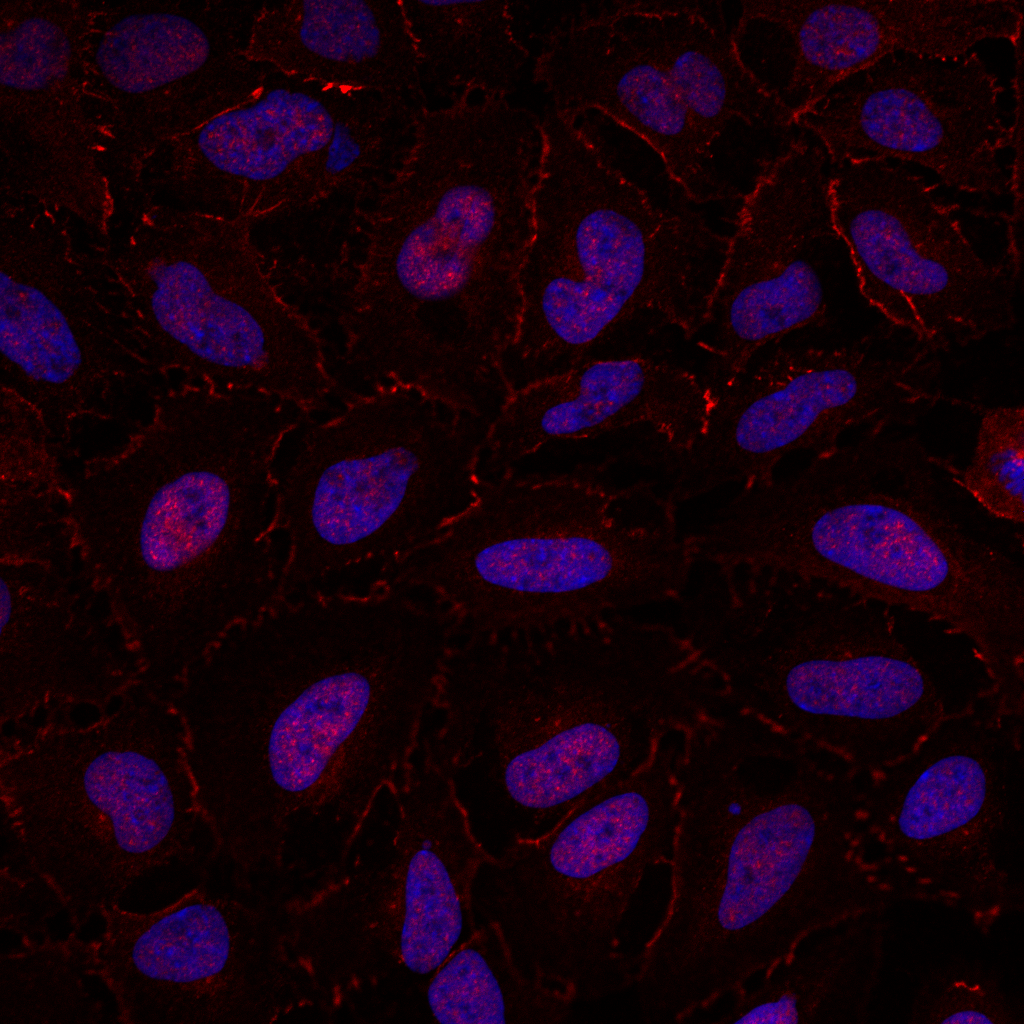

Supplement: Supplementary file 6 [file Data_Sheet_4.ZIP › FIG4 F/control/OCCLUDIN CONTROL.tif]

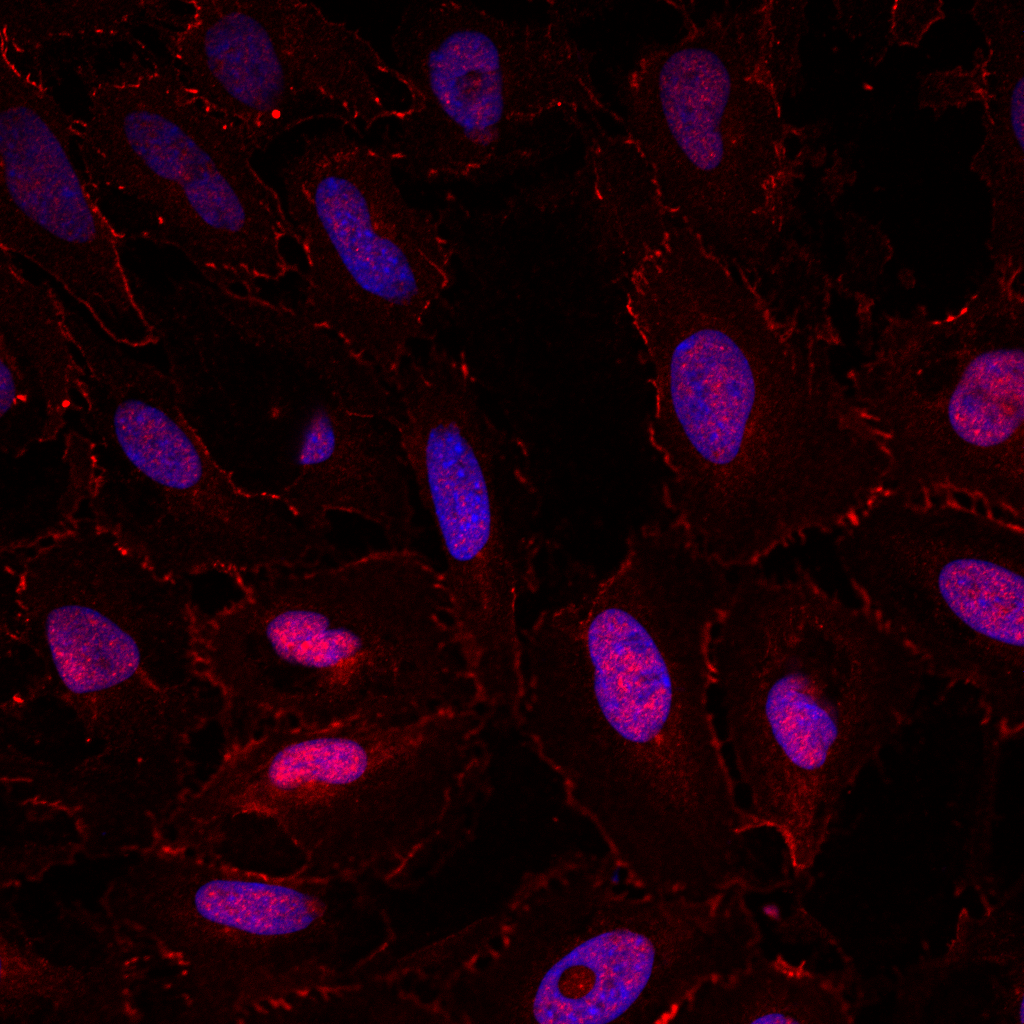

Supplement: Supplementary file 6 [file Data_Sheet_4.ZIP › FIG4 F/control/ZO-1 CONTROL.tif]

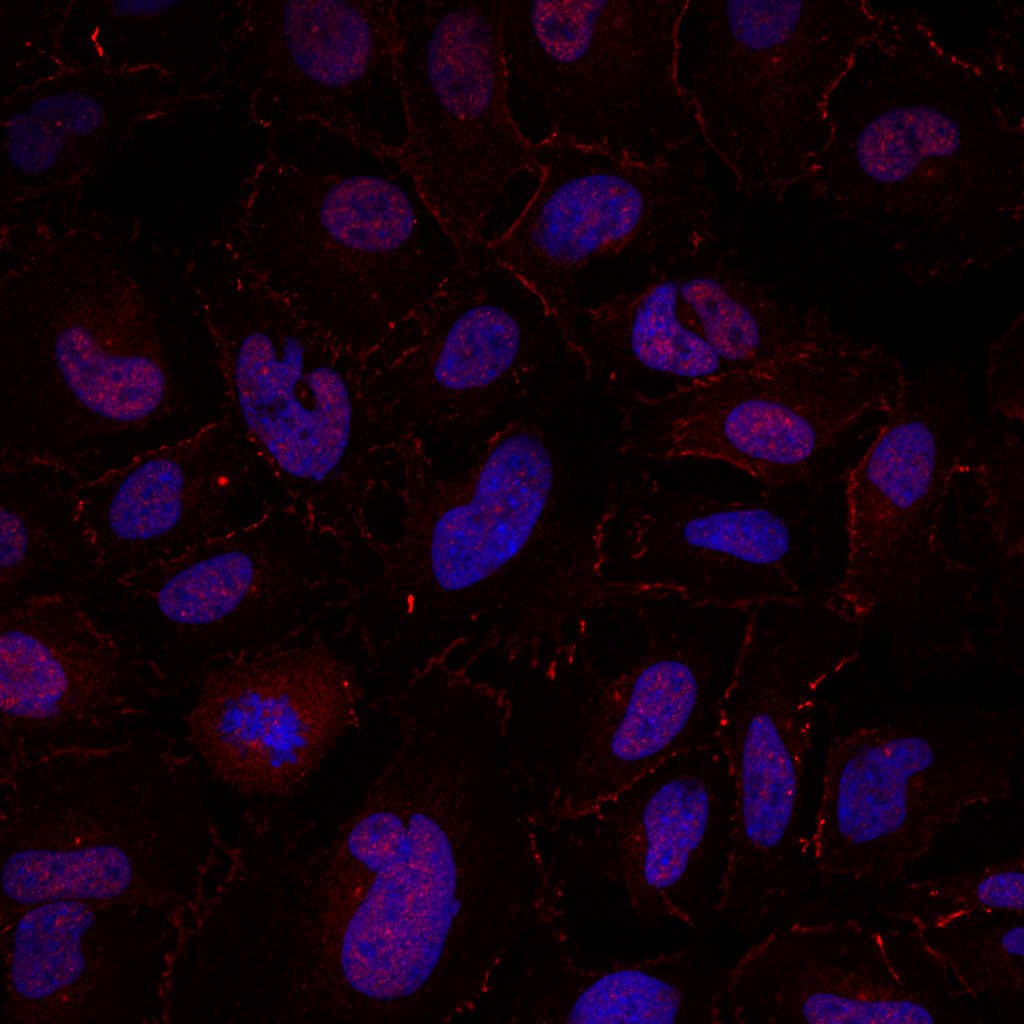

Supplement: Supplementary file 6 [file Data_Sheet_4.ZIP › FIG4 F/HDAC3(-) NC/CLAUDIN5 HDAC3(-) NC.tif]

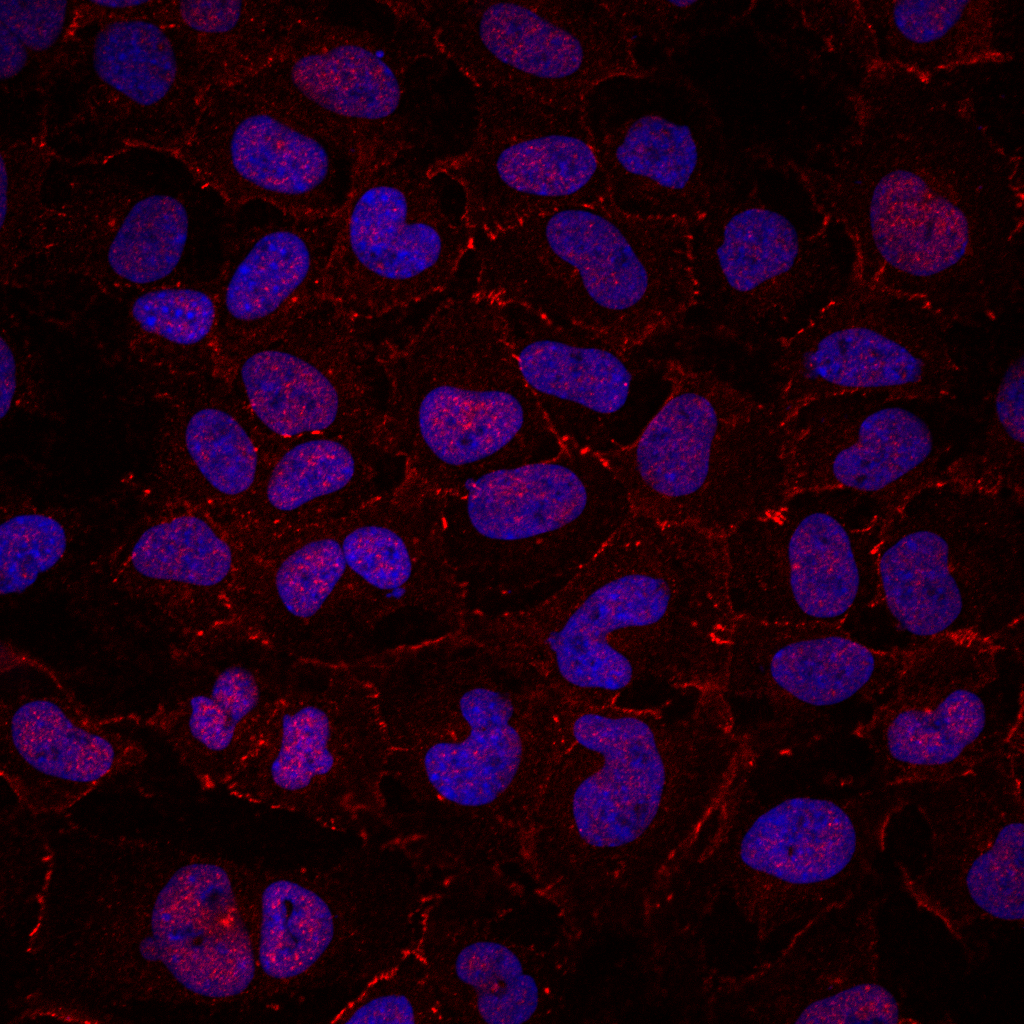

Supplement: Supplementary file 6 [file Data_Sheet_4.ZIP › FIG4 F/HDAC3(-) NC/OCCLUDIN HDAC3(-) NC.tif]

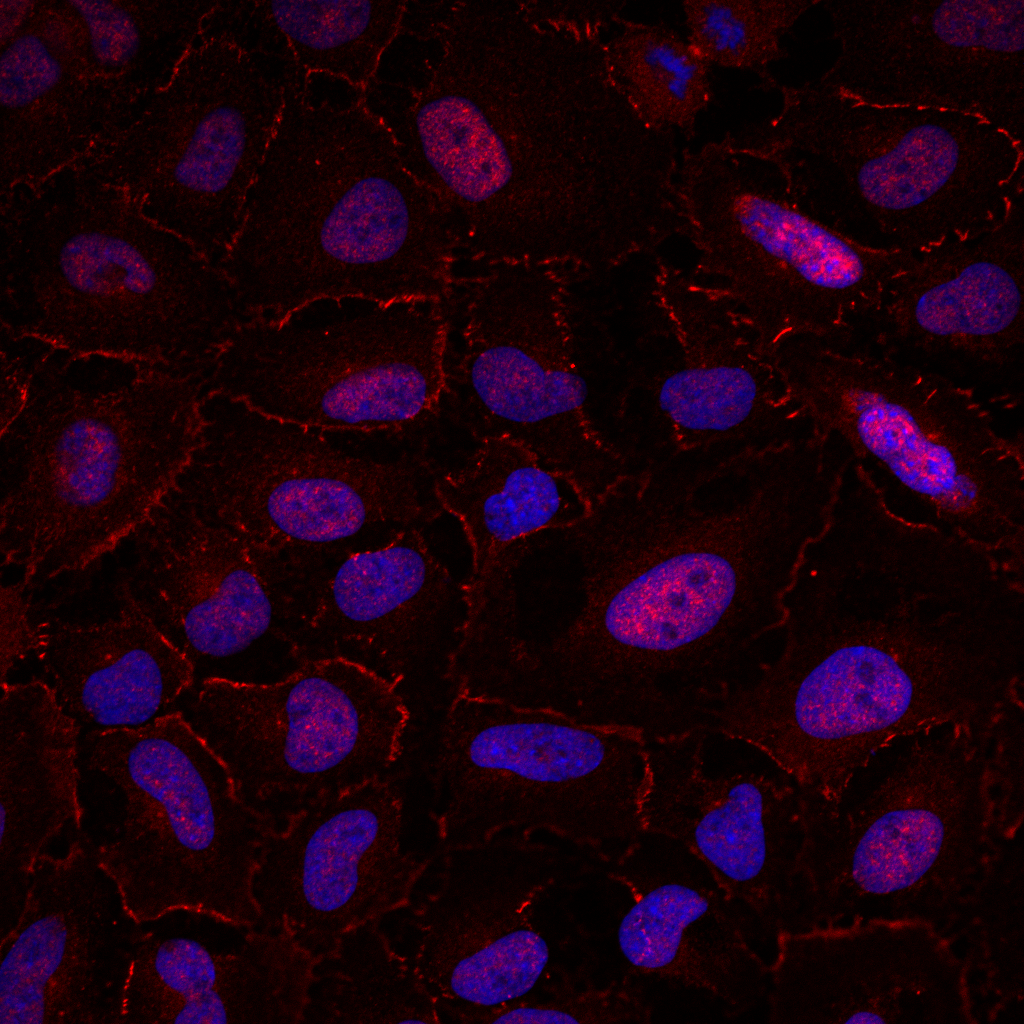

Supplement: Supplementary file 6 [file Data_Sheet_4.ZIP › FIG4 F/HDAC3(-) NC/ZO-1 hdac3(-) NC.tif]

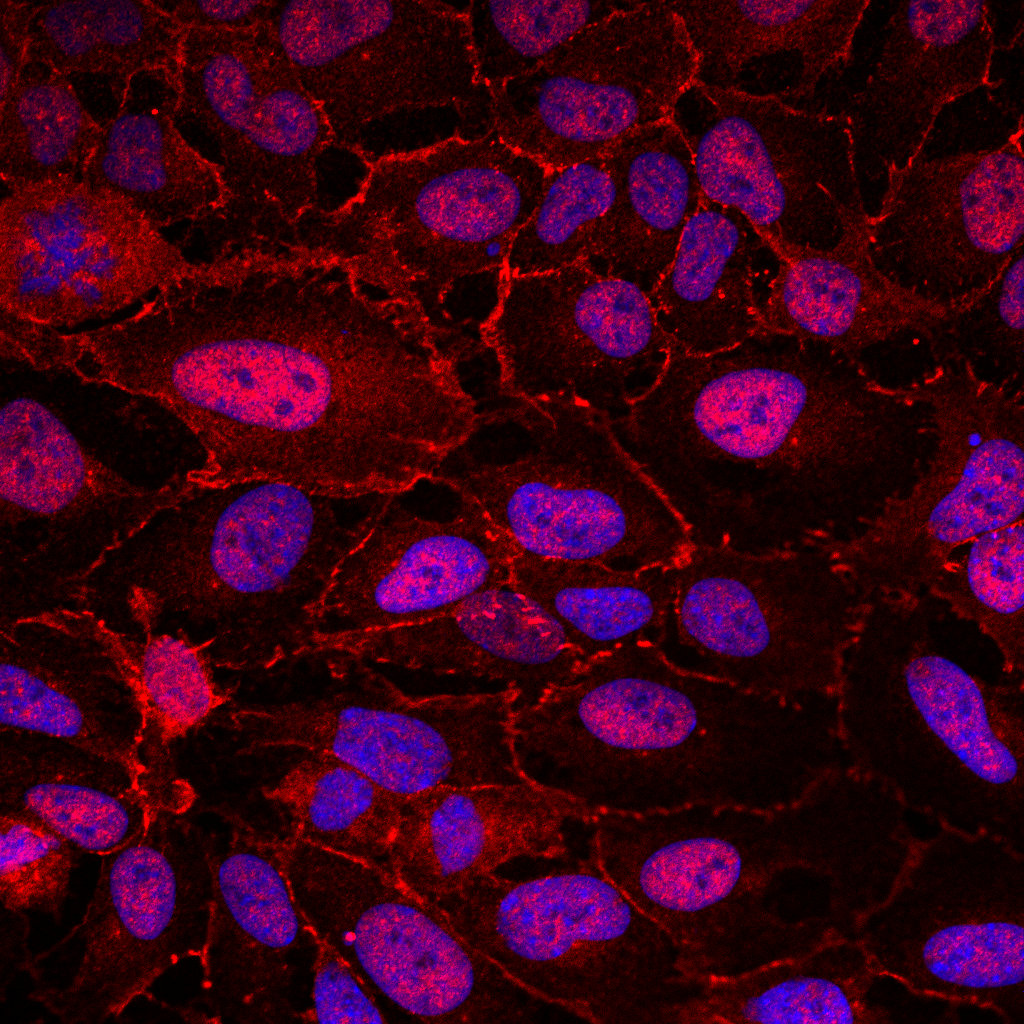

Supplement: Supplementary file 6 [file Data_Sheet_4.ZIP › FIG4 F/HDAC3(-)/CLAUDIN5 hdac3(-).tif]

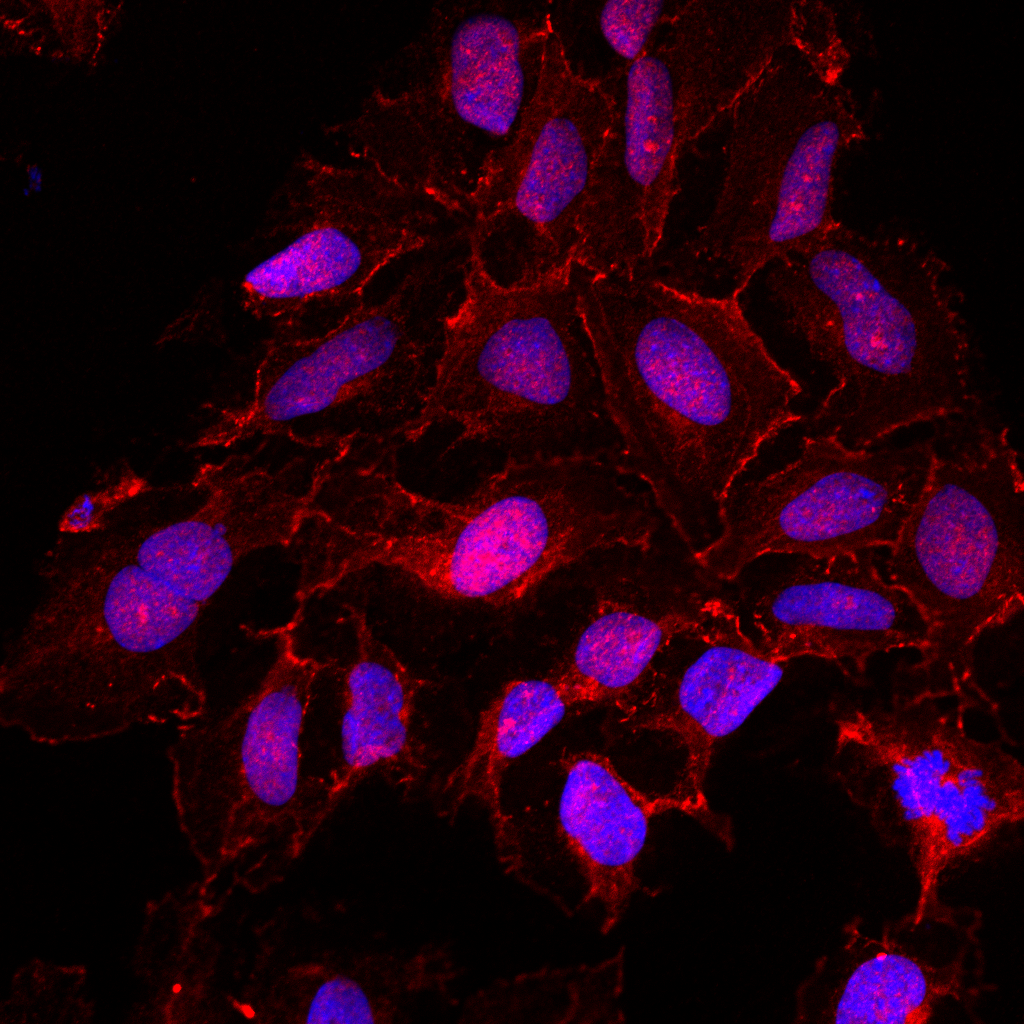

Supplement: Supplementary file 6 [file Data_Sheet_4.ZIP › FIG4 F/HDAC3(-)/OCCLUDIN hdac3(-).tif]

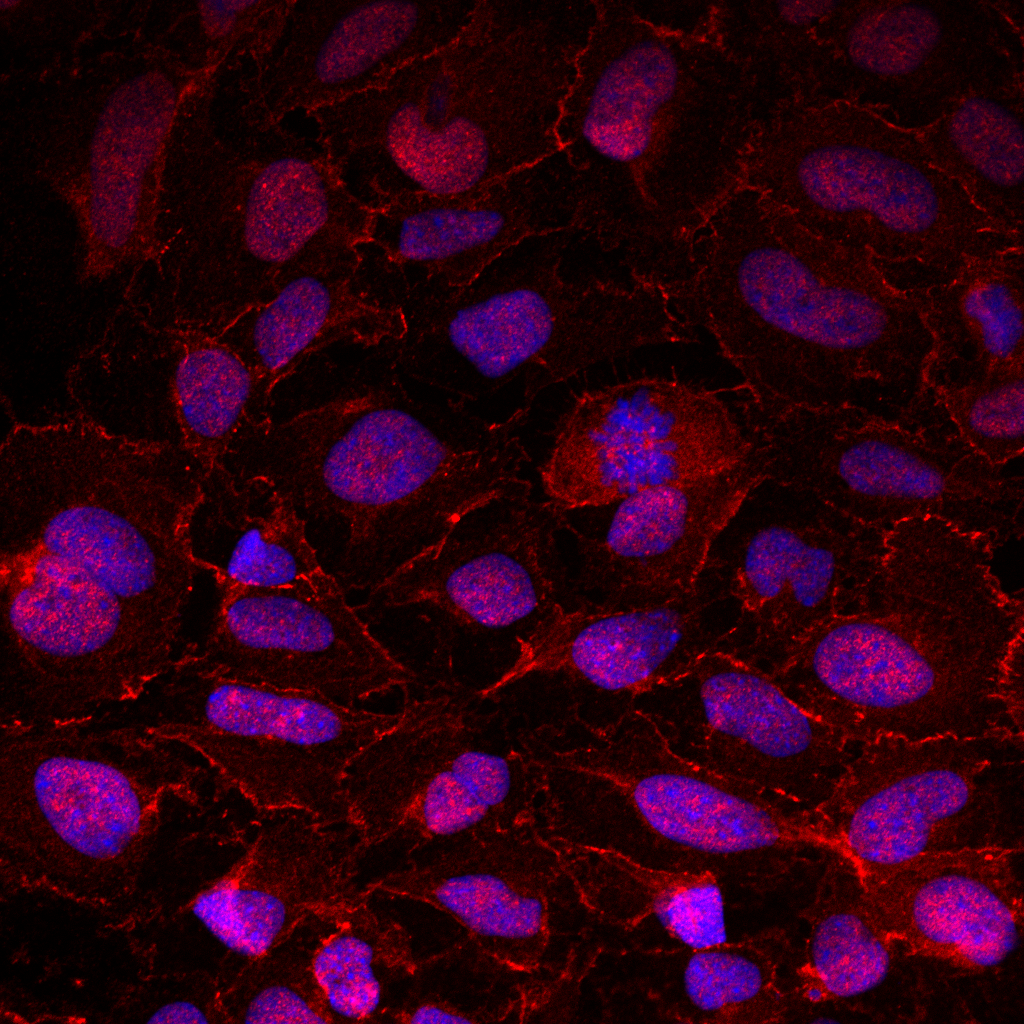

Supplement: Supplementary file 6 [file Data_Sheet_4.ZIP › FIG4 F/HDAC3(-)/ZO-1 hdac3(-).tif]

FIG6 A  
CO-IP of ZNF22

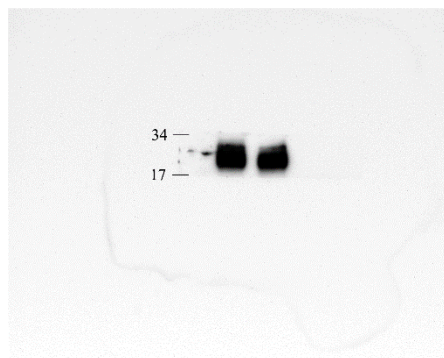

CO-IP of HDAC3

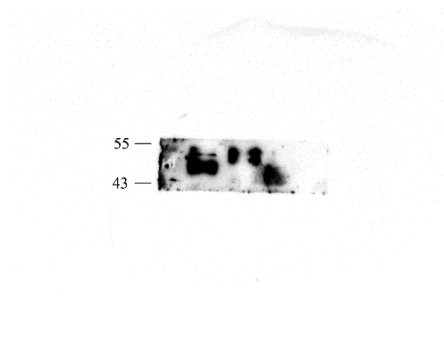

Supplement: Supplementary file 7 [file Data_Sheet_5.ZIP › FIG5 A/FIG5 A Original Western blot pictures.pdf]

FIG6 C

ZNF22 of shHDAC3

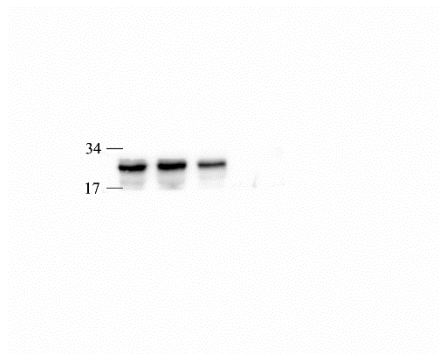

GAPDH of ZNF22 of shHDAC3

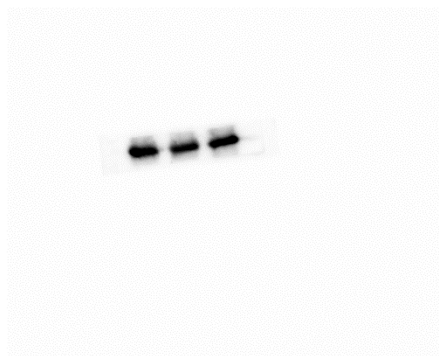

Supplement: Supplementary file 7 [file Data_Sheet_5.ZIP › FIG5 C/FIG5 C Original Western blot pictures.pdf]

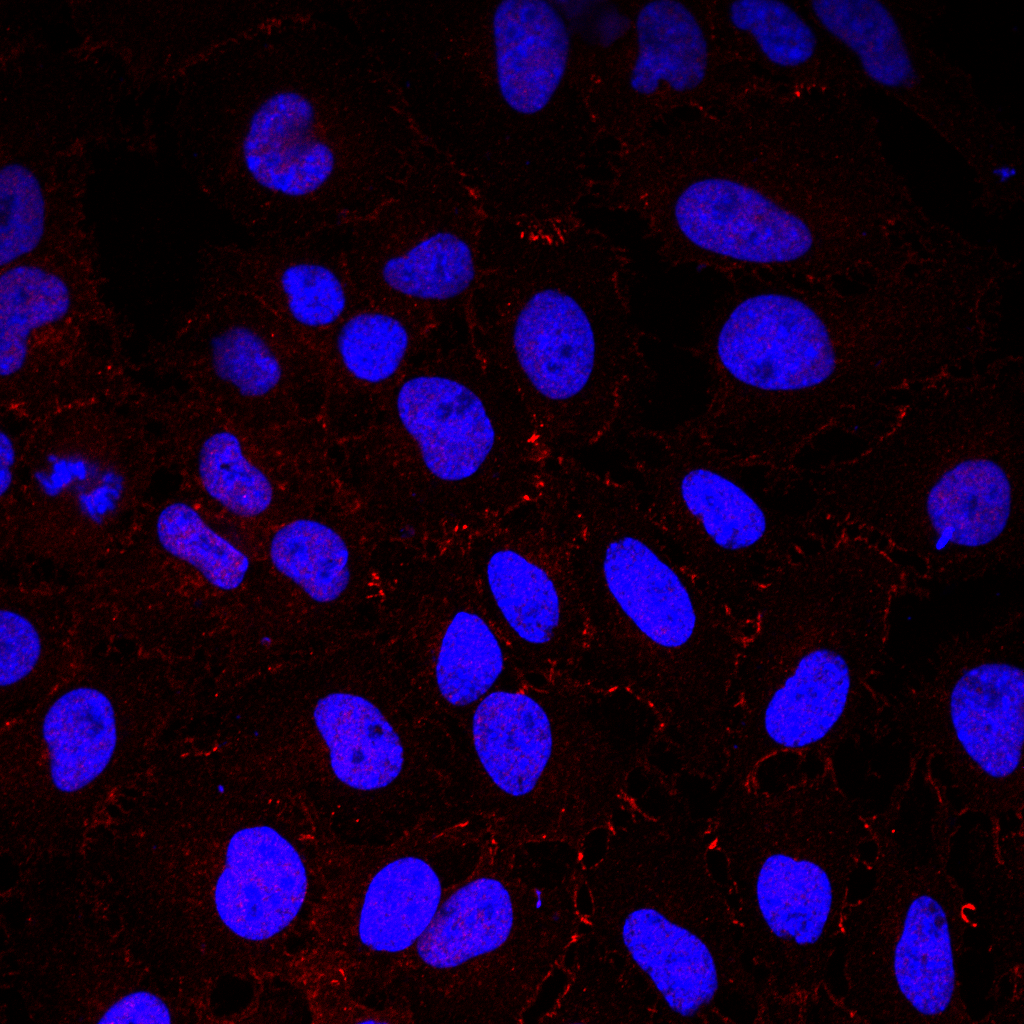

Supplement: Supplementary file 7 [file Data_Sheet_5.ZIP › FIG5 E/control/claudin-5 control.tif]

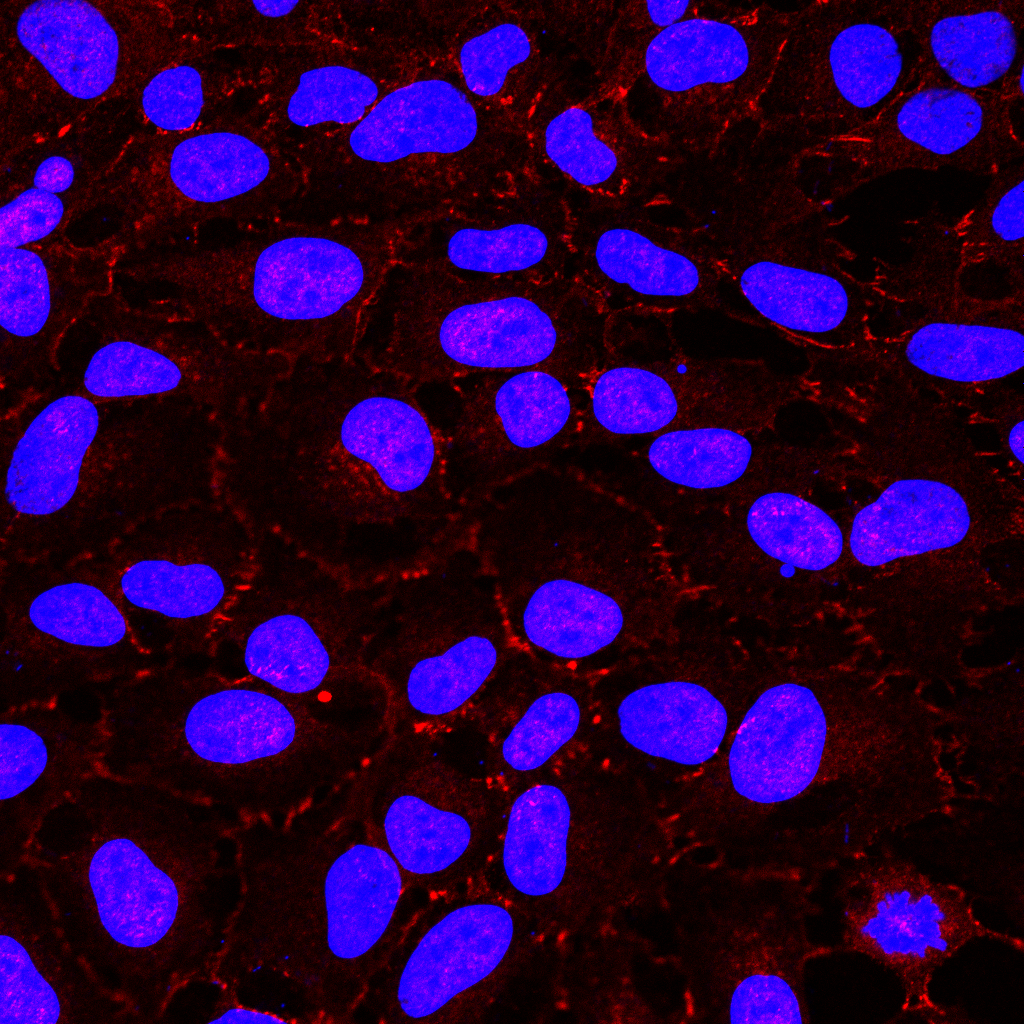

Supplement: Supplementary file 7 [file Data_Sheet_5.ZIP › FIG5 E/control/occludin control.tif]

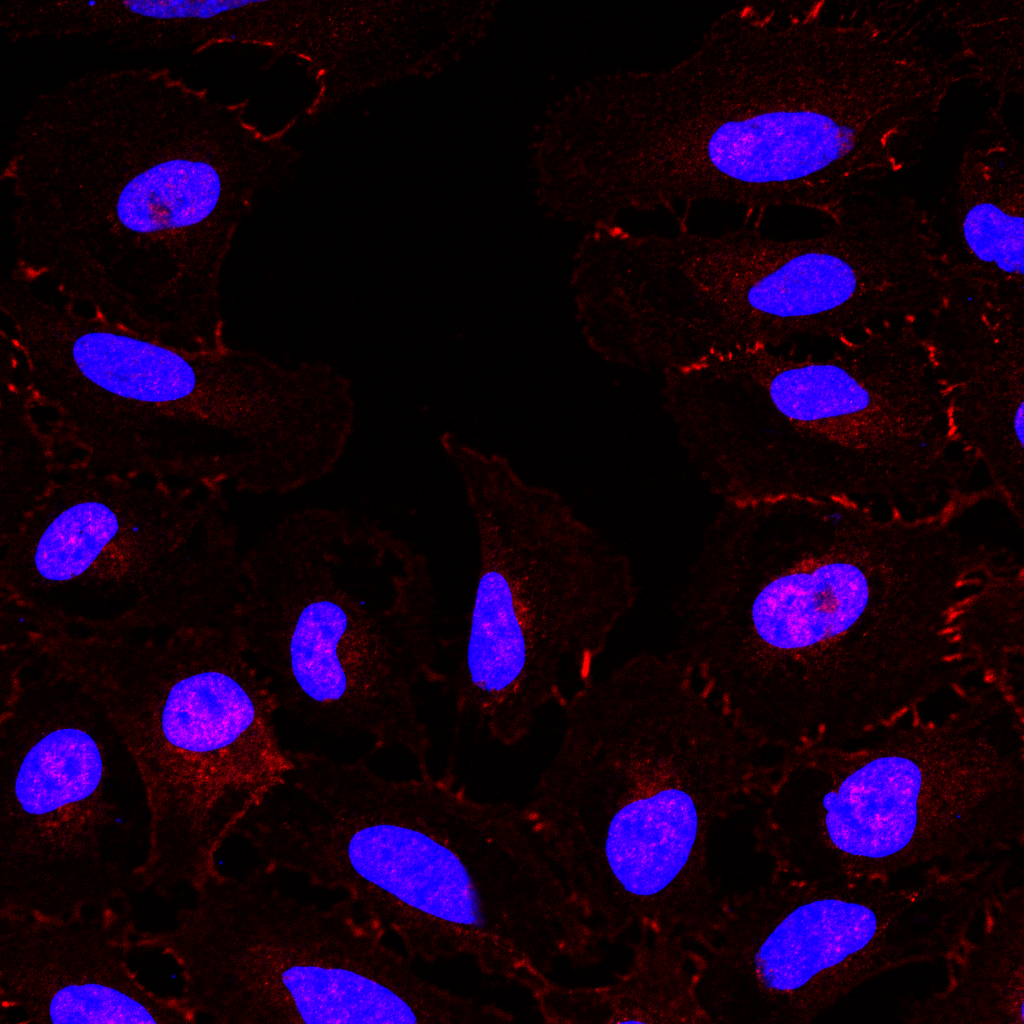

Supplement: Supplementary file 7 [file Data_Sheet_5.ZIP › FIG5 E/control/ZO-1 control.tif]

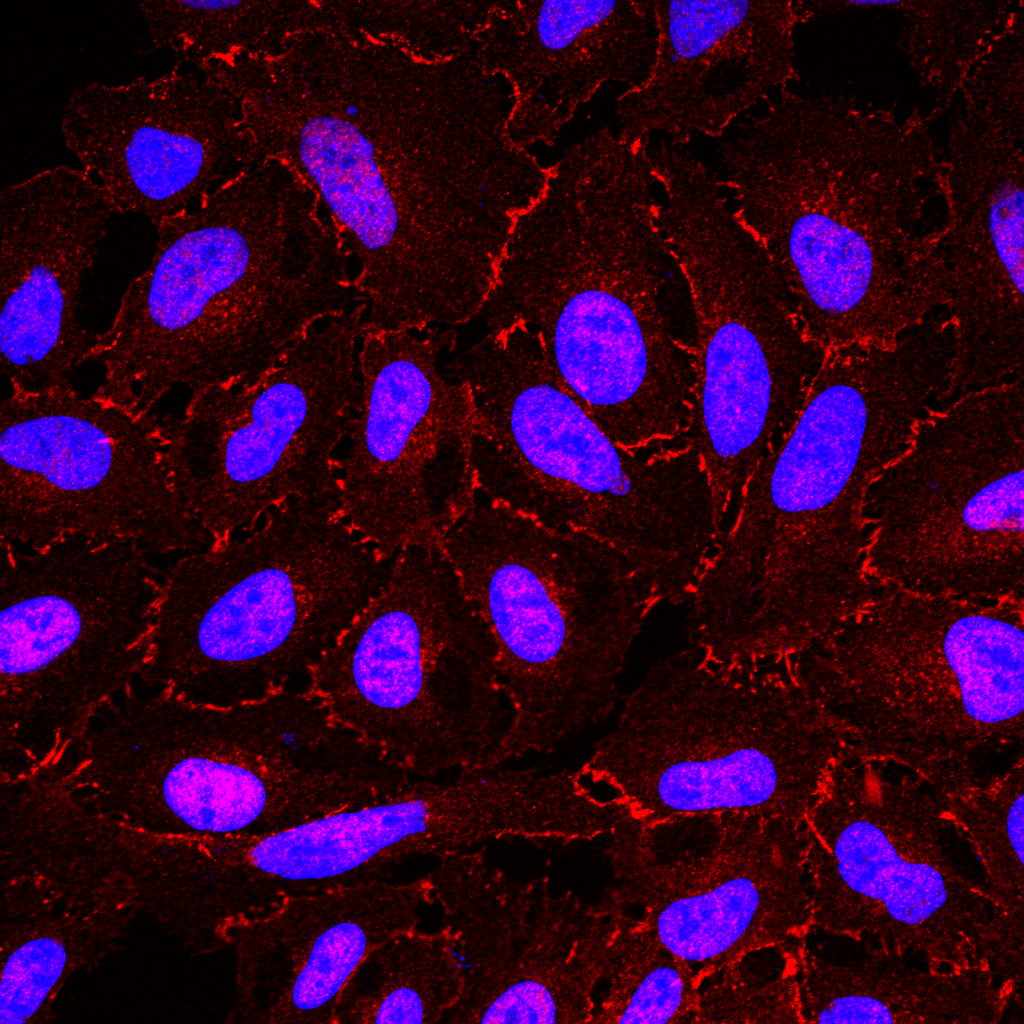

Supplement: Supplementary file 7 [file Data_Sheet_5.ZIP › FIG5 E/HDAC3(-)/claudin-5 hda3c(-).tif]

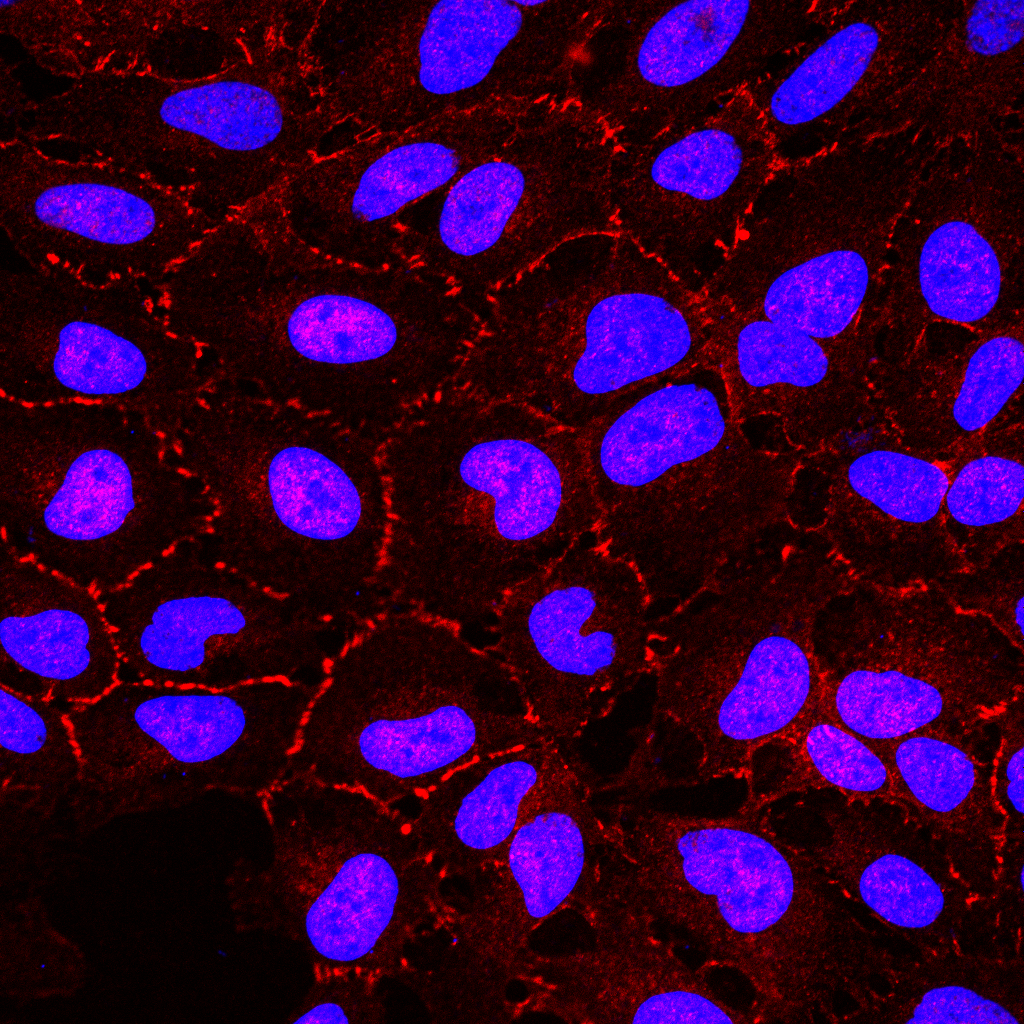

Supplement: Supplementary file 7 [file Data_Sheet_5.ZIP › FIG5 E/HDAC3(-)/occludin hdac3(-).tif]

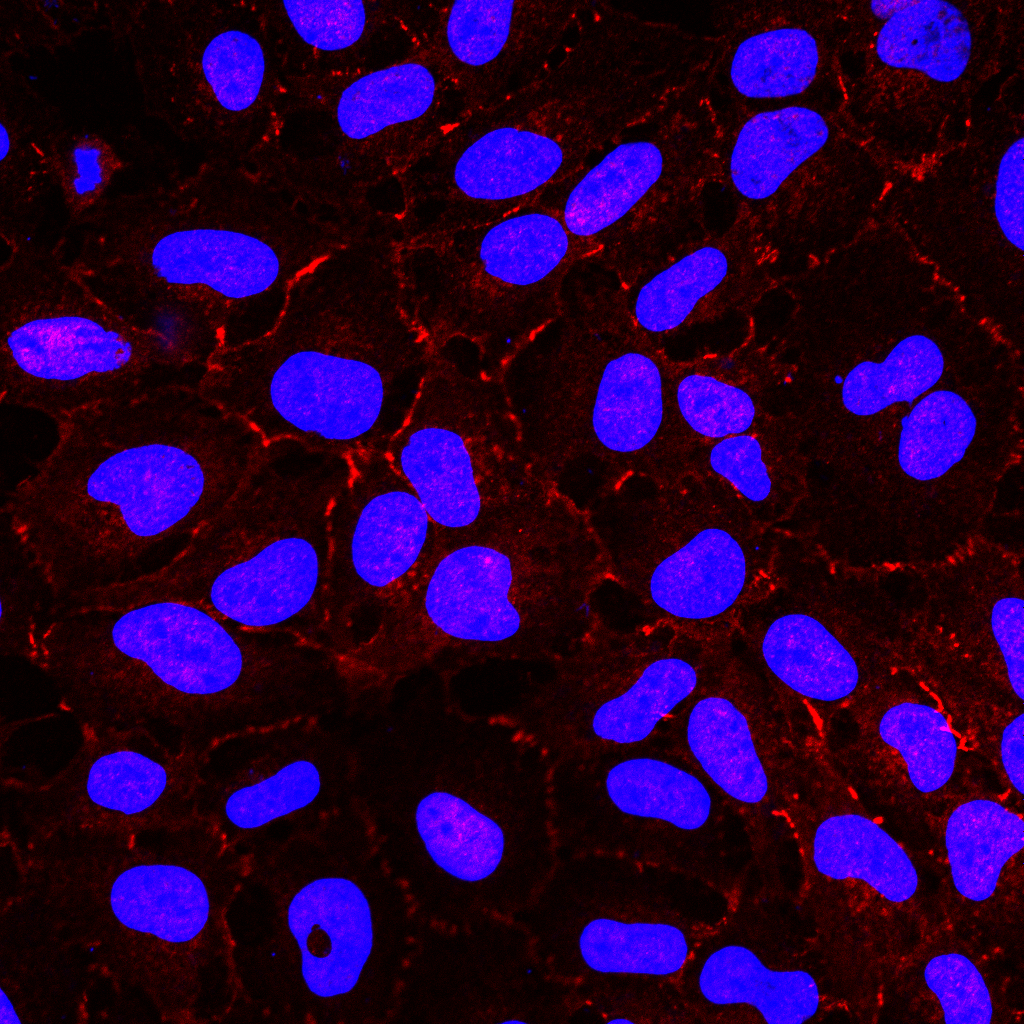

Supplement: Supplementary file 7 [file Data_Sheet_5.ZIP › FIG5 E/HDAC3(-)/ZO-1 HDAC3(-).tif]

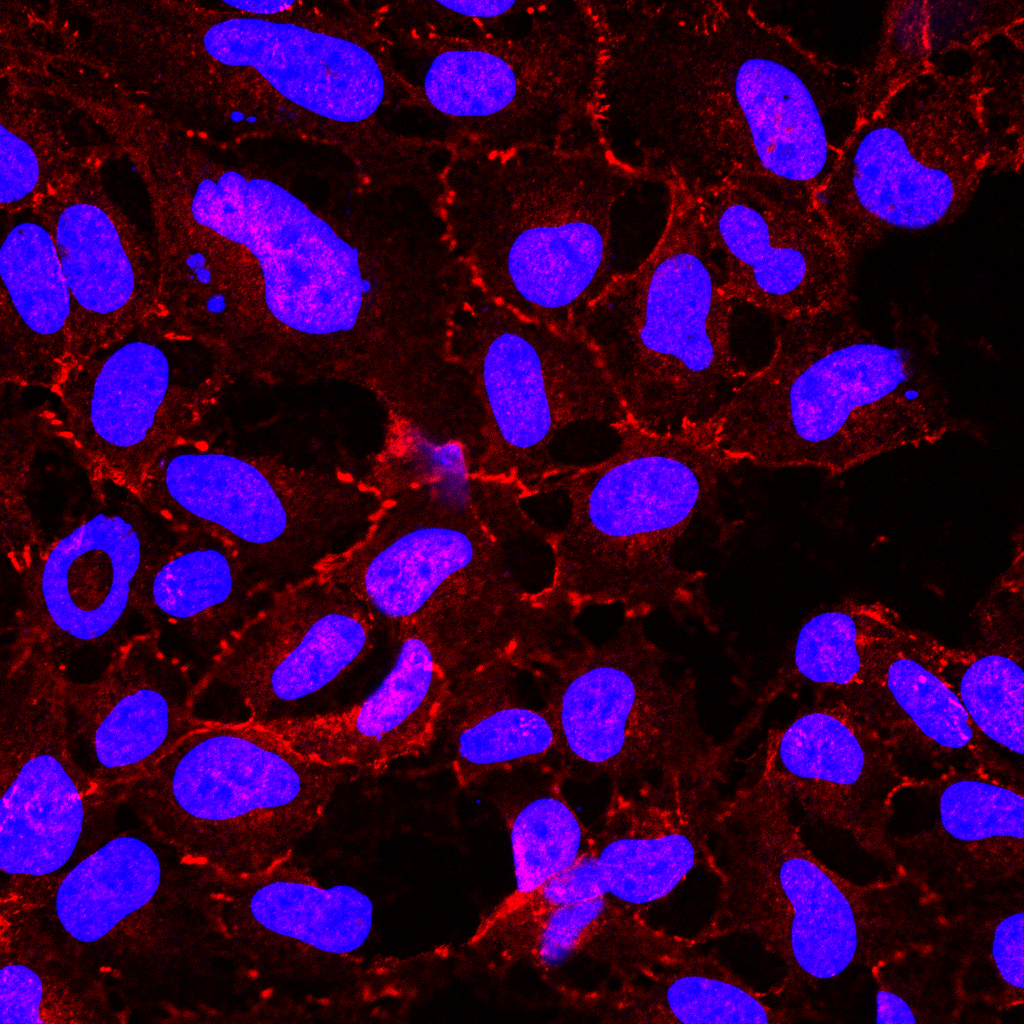

Supplement: Supplementary file 7 [file Data_Sheet_5.ZIP › FIG5 E/HDAC3+ZNF22(-)/cludin5 H+Z(-).tif]

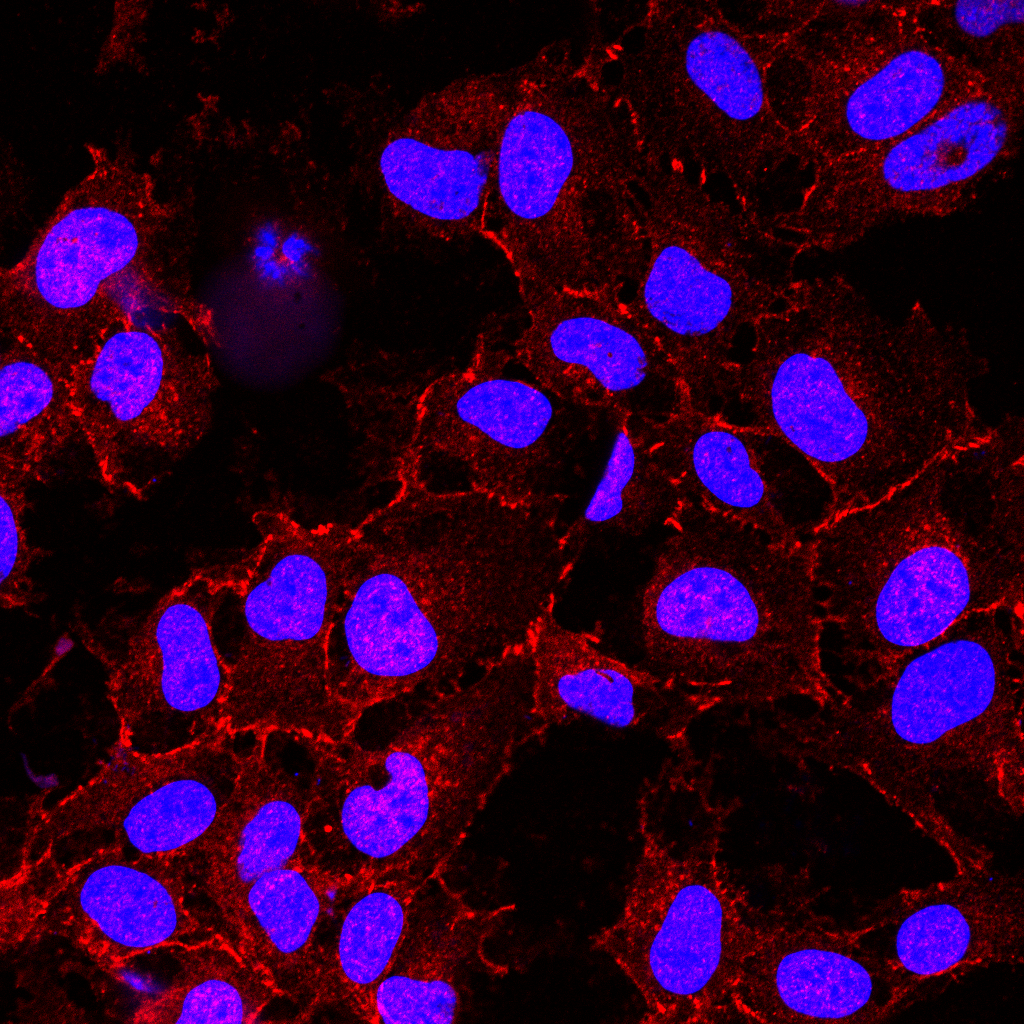

Supplement: Supplementary file 7 [file Data_Sheet_5.ZIP › FIG5 E/HDAC3+ZNF22(-)/occludin H+Z (-).tif]

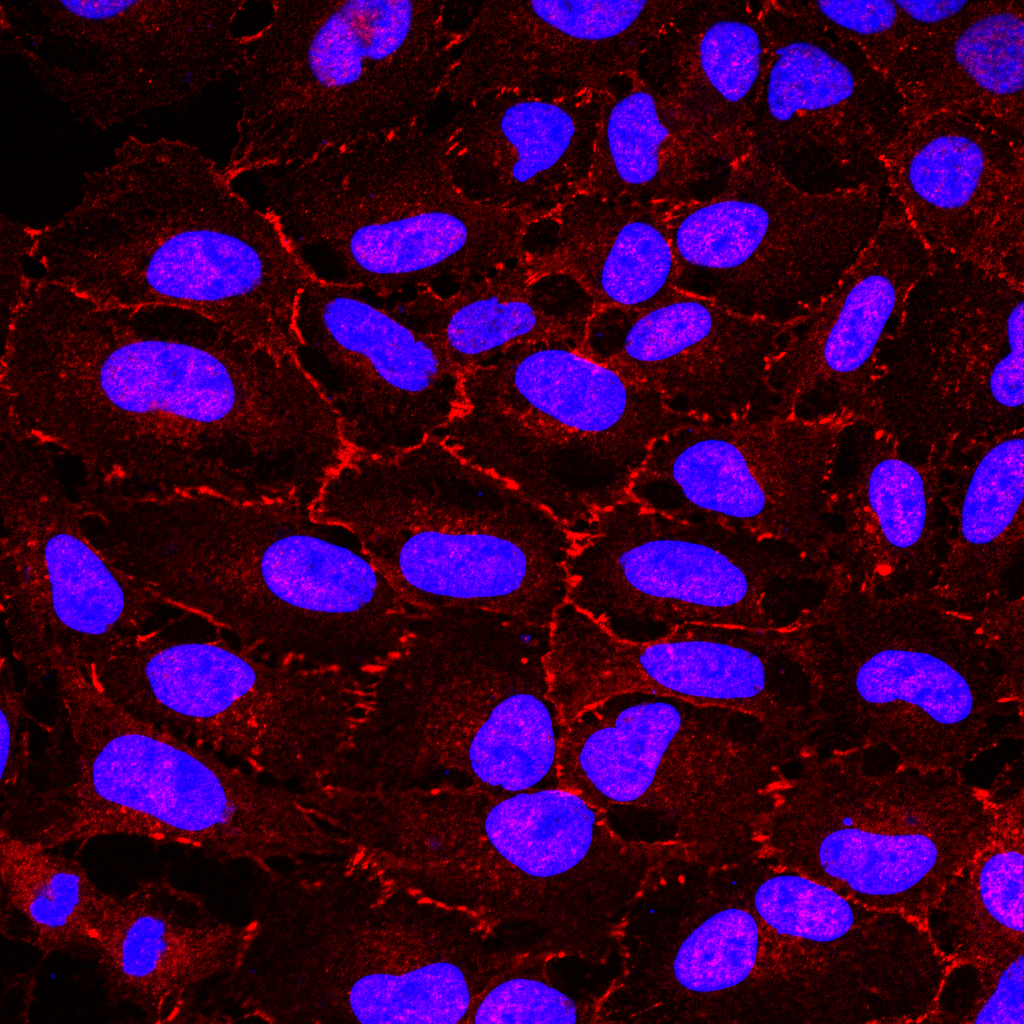

Supplement: Supplementary file 7 [file Data_Sheet_5.ZIP › FIG5 E/HDAC3+ZNF22(-)/ZO-1 H+Z (-).tif]

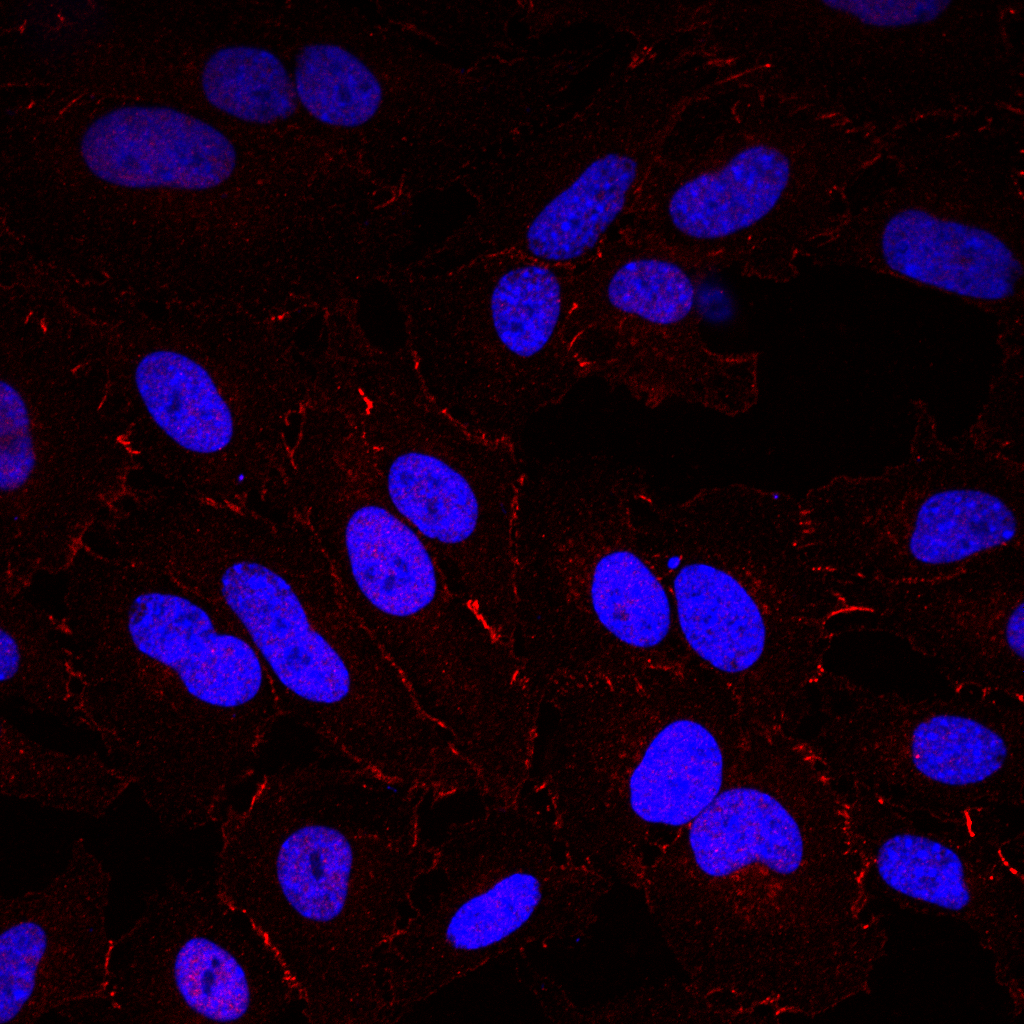

Supplement: Supplementary file 7 [file Data_Sheet_5.ZIP › FIG5 E/NC/cludin shuangNC.tif]

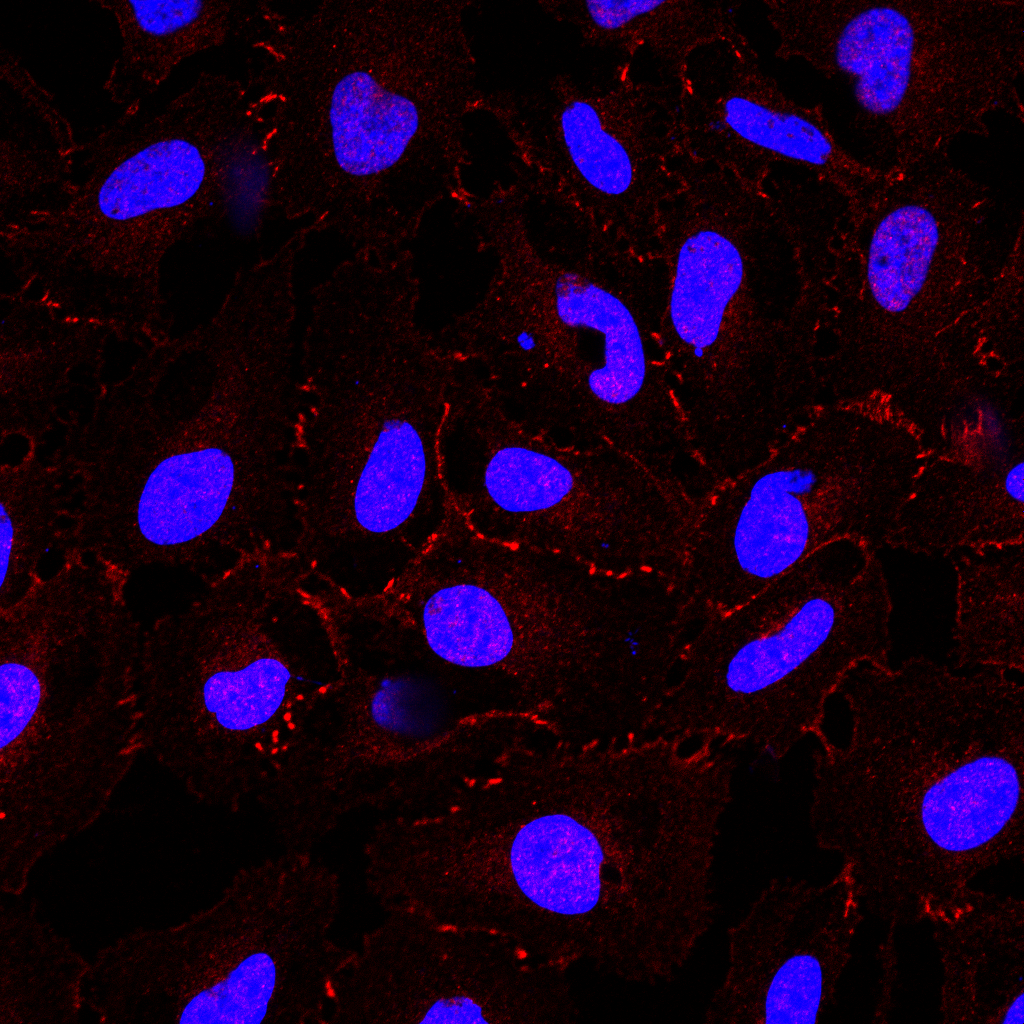

Supplement: Supplementary file 7 [file Data_Sheet_5.ZIP › FIG5 E/NC/occlu nc.tif]

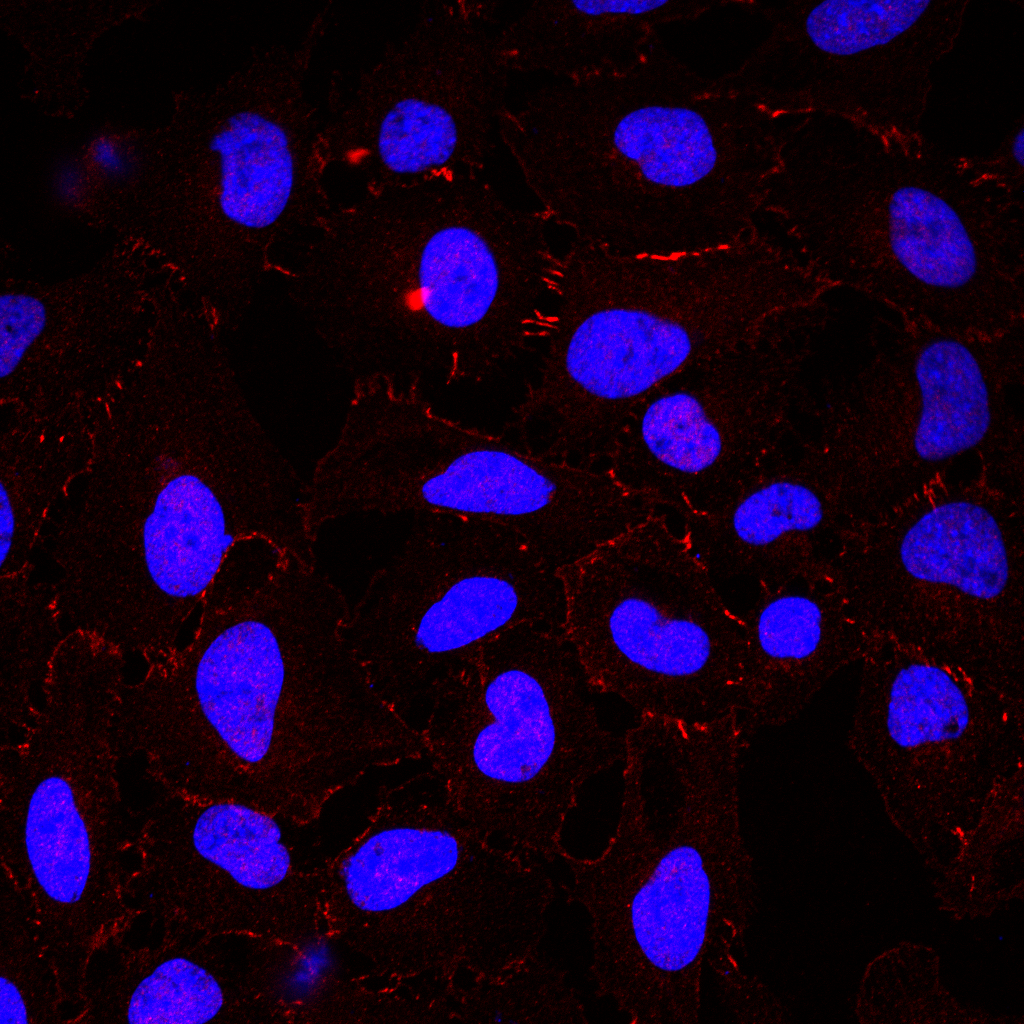

Supplement: Supplementary file 7 [file Data_Sheet_5.ZIP › FIG5 E/NC/zo1-2NC.tif]

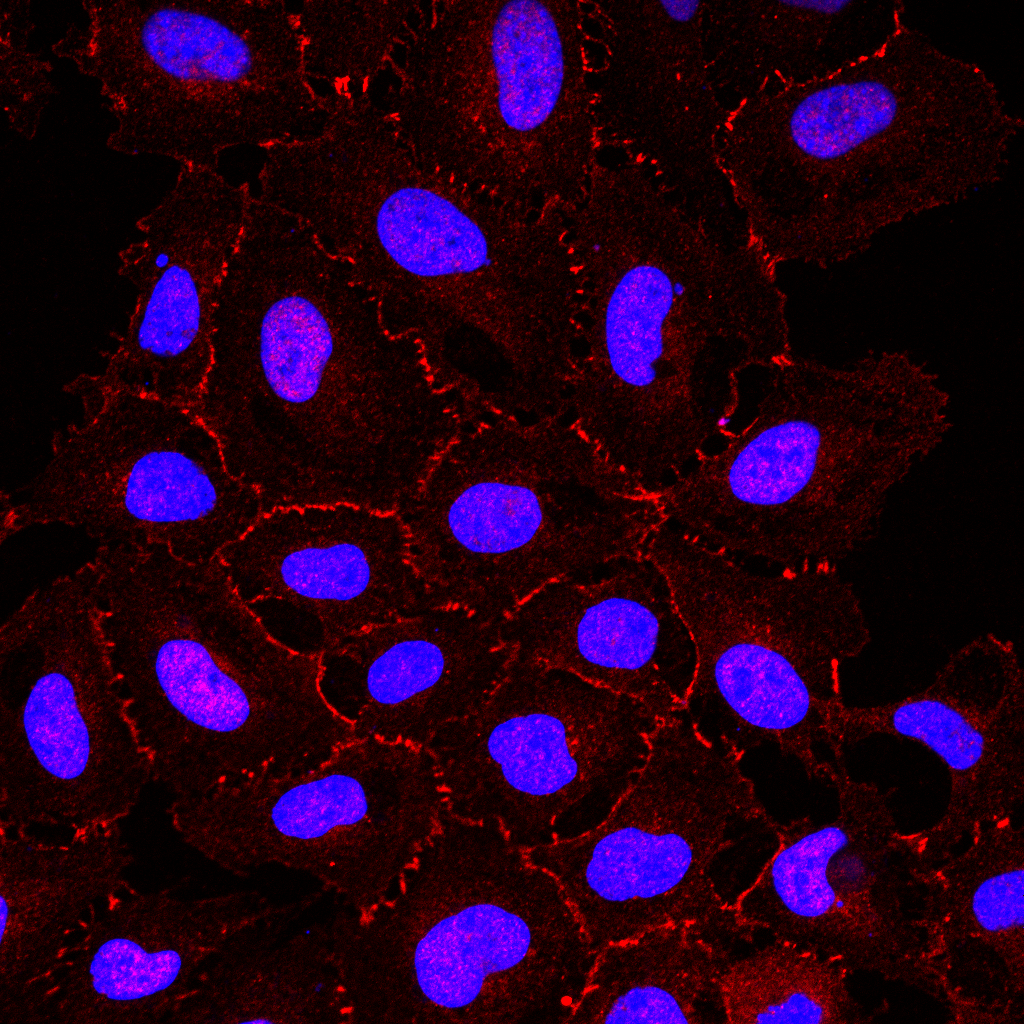

Supplement: Supplementary file 7 [file Data_Sheet_5.ZIP › FIG5 E/ZNF22(-)/cludin znf22-.tif]

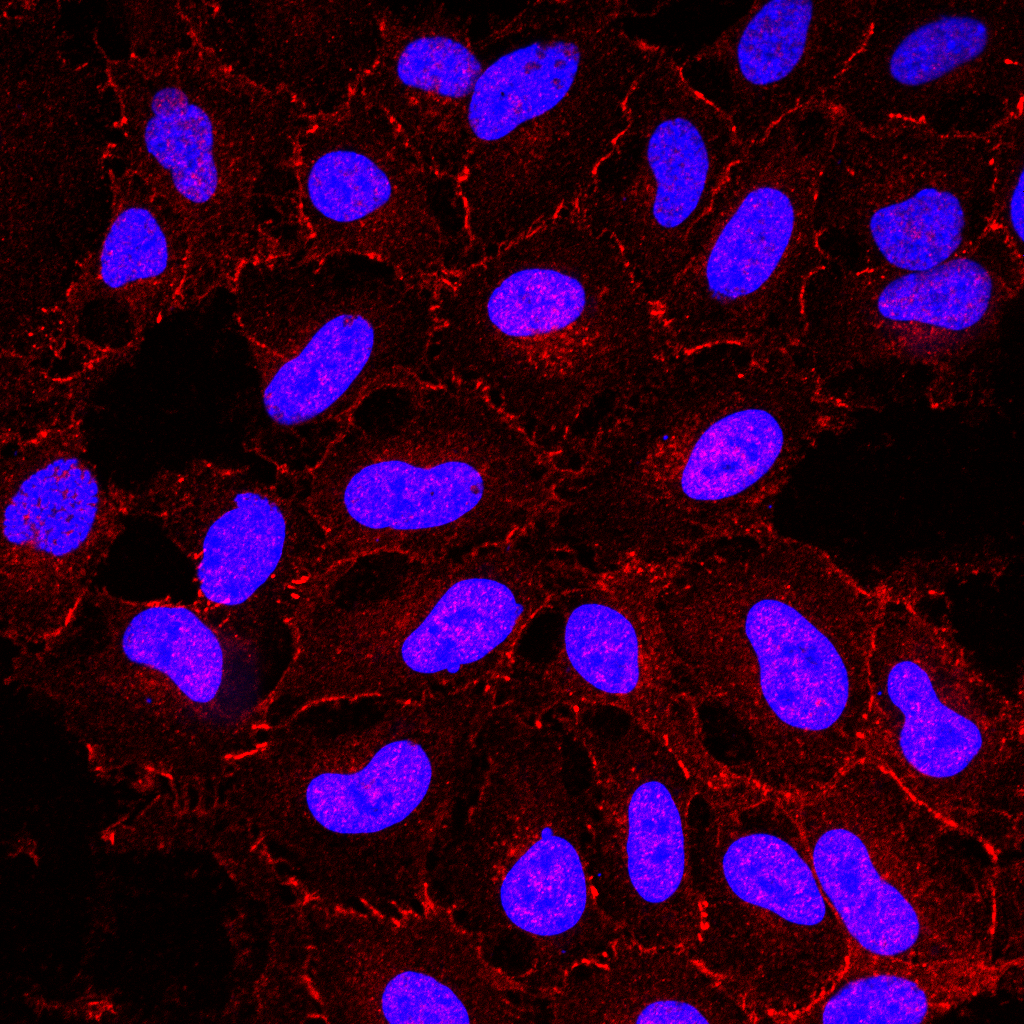

Supplement: Supplementary file 7 [file Data_Sheet_5.ZIP › FIG5 E/ZNF22(-)/occlu znf22-.tif]

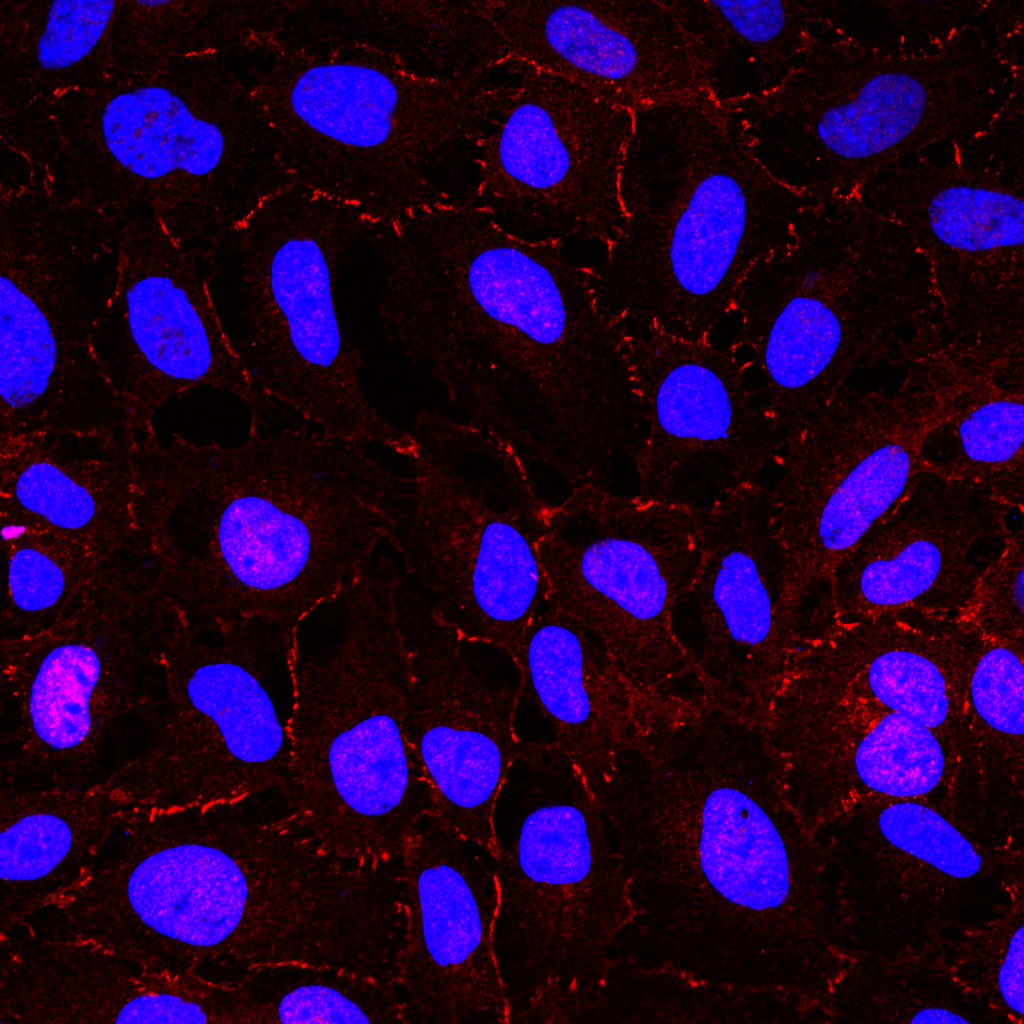

Supplement: Supplementary file 7 [file Data_Sheet_5.ZIP › FIG5 E/ZNF22(-)/zo-1ZNF22(-).tif]
