# Supplementary material for: Transcription factor ZNF22 regulates blood-tumor barrier permeability by interacting with HDAC3 protein
Source: Front Mol Neurosci. 2022 Nov 28;15:1027942. doi: 10.3389/fnmol.2022.1027942 (PMC9742255; doi:10.3389/fnmol.2022.1027942)
Supplement: Supplementary file 4 [file Data_Sheet_2.ZIP › FIG2 B/FIG3B Original Western blot pictures.pdf]

Relative expression of ZNF22

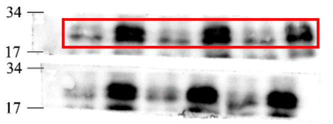

GAPDH of relative expression of ZNF22

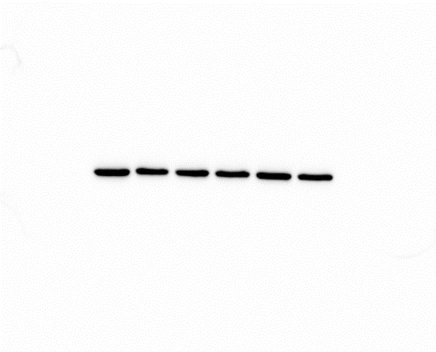

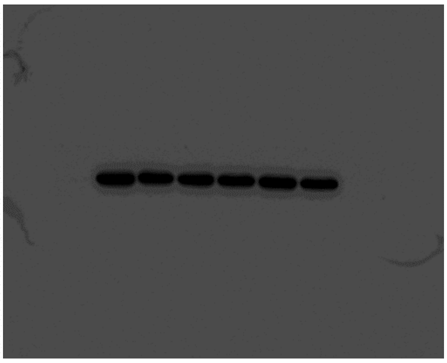

The original western blot pictures are shown. Because of the characteristics of monoclonal antibodies we used, the background of the ECL luminescence of some proteins was relatively clean. Therefore, the GAPDH of relative expression of ZNF22 colour levels were adjusted to show the imprint of the peripheral ECL substrate more clearly, and the image is presented on the right. In other words, we provided two different backgrounds of the same image to prove that we had not done any cropping to the original image.
